# Supplementary material for: Expression of ALS-PFN1 impairs vesicular degradation in iPSC-derived microglia
Source: Nat Commun. 2024 Mar 20;15:2497. doi: 10.1038/s41467-024-46695-w (PMC10954694; doi:10.1038/s41467-024-46695-w)
Supplement: Supplementary file 1 — Supplementary Information [file 41467_2024_46695_MOESM1_ESM.pdf]

SUPPLEMENTARY FIGURES

a

	Code	Line Name	Genotype
C71G-PFN1 isogenic group	WT	KOLF2.1J: PFN1_C71G WT clone B03	WT clone during C71G mutation editing
	C71G$^{+/-}$	KOLF2.1J: PFN1_C71G het clone C01	C71G/WT
M114T-PFN1 isogenic group	WT	KOLF2.1J: PFN1_M114T WT clone H09	WT clone during M114T mutation editing
	M114T$^{+/-}$	KOLF2.1J: PFN1_M114T het clone A08	M114T/WT
	M114T$^{+/+}$	KOLF2.1J: PFN1_M114T homo clone D12	M114T/M114T

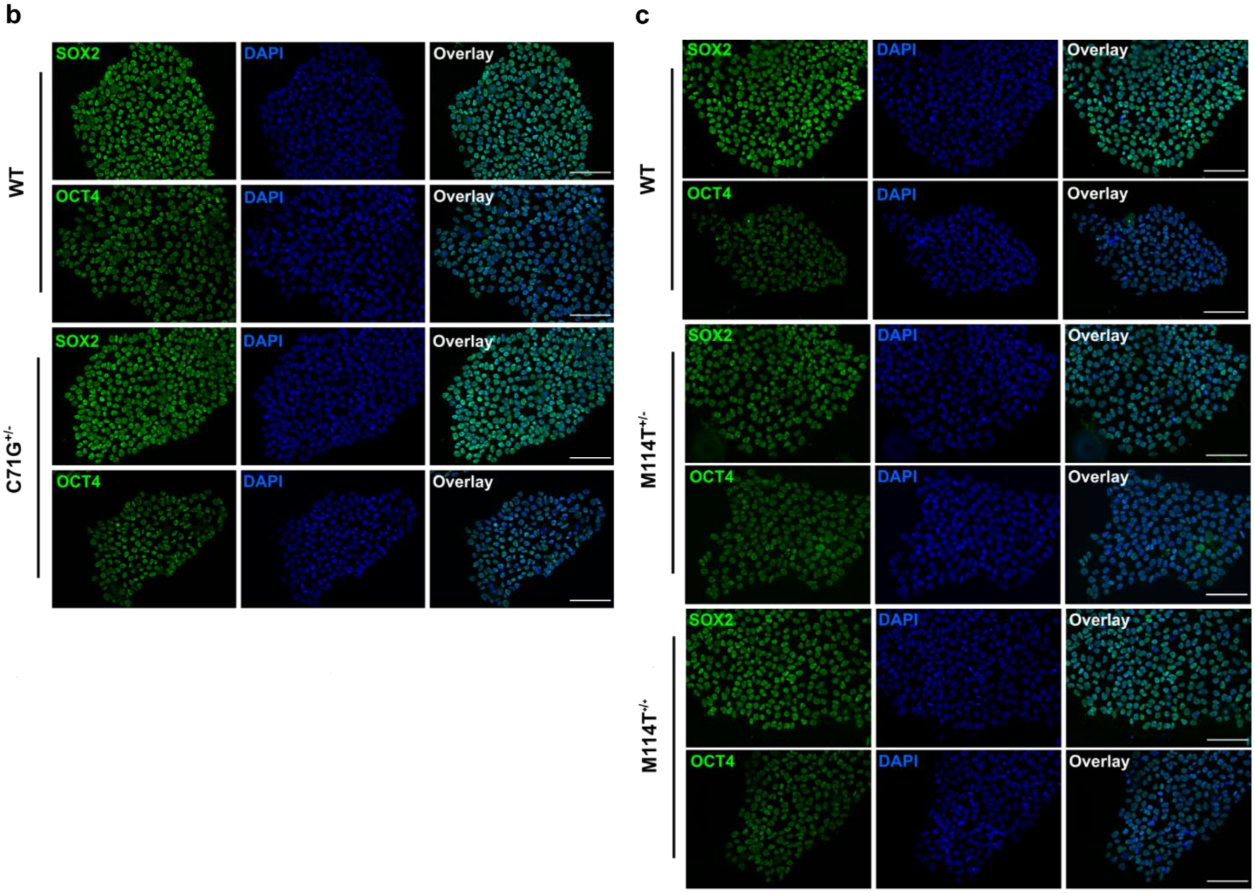

Supplemental Figure 1. Pluripotency analysis of iPSC lines used in this study. a, iPSC lines used in this report. **b-c,** Immunofluorescence images of pluripotency markers SRY-Box transcription factor 2 (SOX2; green) and octamer-binding transcription factor 4 (OCT4; green) in WT and C71G$^{+/-}$ iPSCs (**b**) and WT, M114T$^{+/-}$ and M114T$^{+/+}$ iPSCs (**c**). Representative images obtained from one analysis. Scale bar: 100 µm.

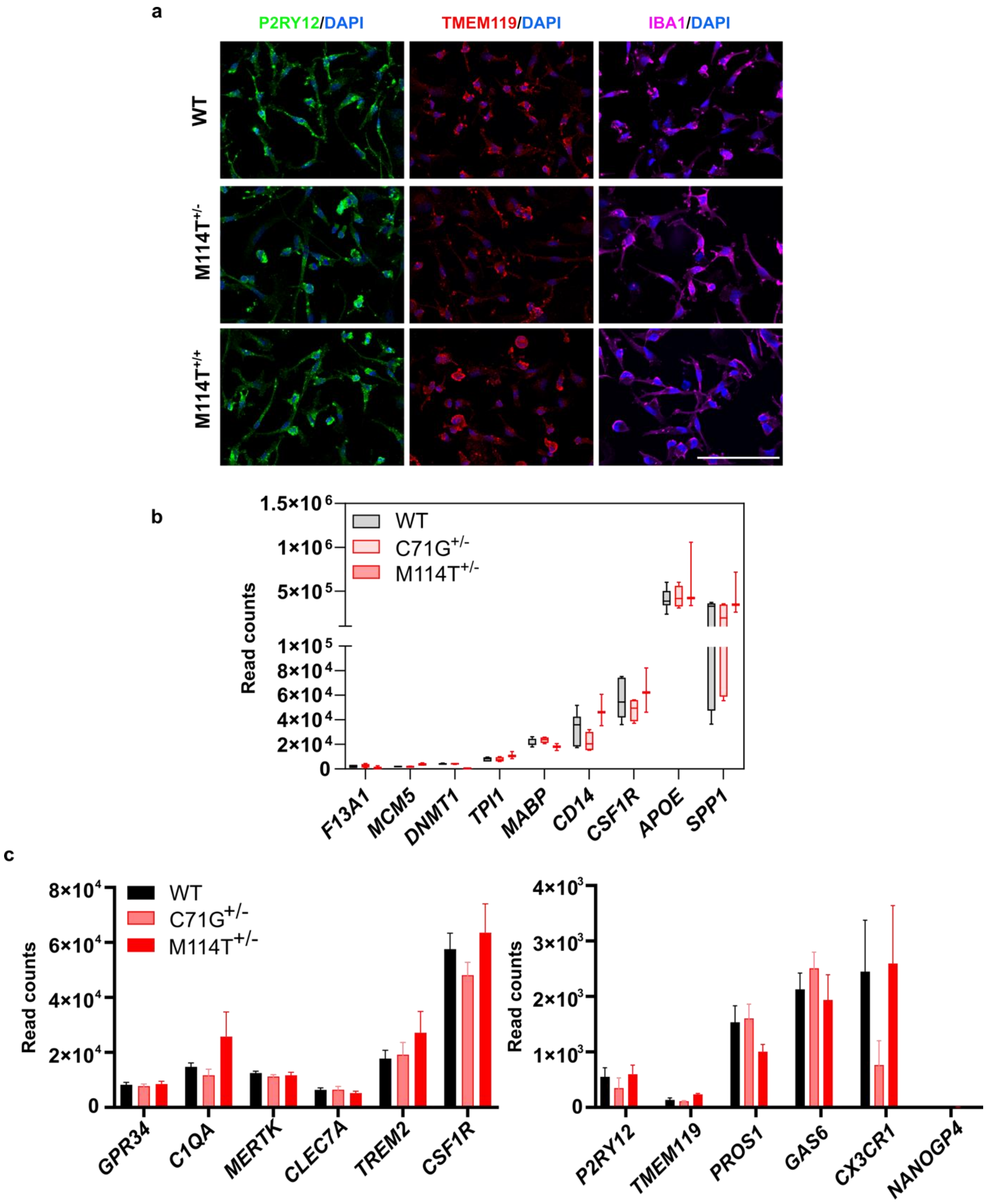
a
P2RY12/DAPI
TMEM119/DAPI
IBA1/DAPI
WT
M114T+/-
M114T+/+
b
WT
C71G+/-
M114T+/-
Read counts
1.5×10^6
1×10^6
5×10^5
1×10^5
8×10^4
6×10^4
4×10^4
2×10^4
0
F13A1
MCM5
DNMT1
TPI1
MABP
CD14
CSF1R
APOE
SPP1
c
WT
C71G+/-
M114T+/-
Read counts
8×10^4
6×10^4
4×10^4
2×10^4
0
GPR34
C1QA
MERTK
CLEC7A
TREM2
CSF1R
4×10^3
3×10^3
2×10^3
1×10^3
0
P2RY12
TMEM119
PROS1
GAS6
CX3CR1
NANOGP4

Supplemental Figure 2. Expression of microglia and myeloid markers in ALS-PFN1 and isogenic control iMGs. a, Representative immunofluorescence images of the microglia and myeloid markers P2RY12, transmembrane protein 119 (TMEM119), and ionized calcium-binding adapter molecule 1 (IBA1) in WT, M114T$^{+/-}$ and M114T$^{+/+}$ iMGs. Scale bar: 100 µm. **b,** Normalized read counts of genes associated with different microglia developmental stages expressed in mutant PFN1 and WT iMGs, including early-stage yolk-sac microglia progenitor gene *F13A1* and embryonic-microglia genes minichromosome maintenance complex component 5 (*MCM5*), DNA methyltransferase 1 (*DNMT1*), and triosephosphate isomerase 1 (*TPI1*) are shown. Also shown are adult-microglia genes V-Maf musculoaponeurotic fibrosarcoma oncogene homolog B (*MAFB*), monocyte differentiation antigen CD14 (*CD14*) and colony-stimulating factor-1 receptor *(CSFR1)*, and the aging-related microglia genes Apolipoprotein E (*APOE)* and secreted phospho-protein 1 (*SPP1).* Box and whisker plots of WT n=7, C71G$^{+/-}$ n=4, and M114T$^{+/-}$ n=3 independent differentiations. **c,** Normalized read counts of the microglia-enriched genes *GPR34*, complement C1q A Chain (*C1QA*), *MERTK*, C-type lectin domain containing 7A (*CLEC7A*), triggering receptor expressed on myeloid cells 2 (*TREM2*), CSF1 receptor (*CSF1R*), *P2RY12*, *TMEM119*, *PROS1*, growth arrest specific 6 (*GAS6*), C-X3-C motif chemokine receptor 1 (*CX3CR1*), and the stem-cell related gene Nanog homeobox pseudogene 4 (*NANOGP4*) expressed in mutant PFN1 and WT iMGs. Bar graphs show mean ± SEM for WT n=7, C71G$^{+/-}$ n=4, and M114T$^{+/-}$ n=3 independent differentiations. Source data are provided as a Source Data file.

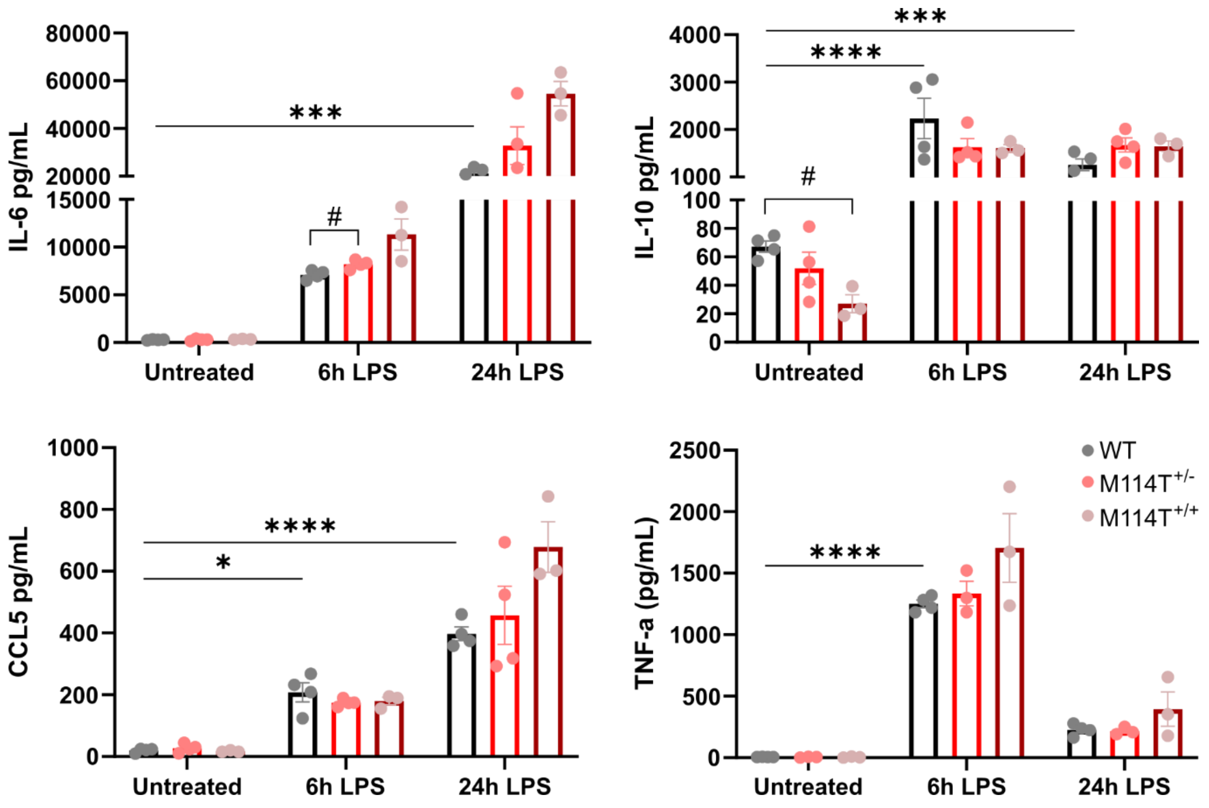

Supplemental Figure 3. Cytokine secretion upon LPS stimulation in PFN1 M114T$^{+/-}$ and M114T$^{+/+}$ iMGs. WT, M114T$^{+/-}$, and M114T$^{+/+}$ iMGs secrete elevated levels of IL-6 (****P*=0.0003), IL-10 (****P*=0.0003, *****P*<0.0001), CCL5 (**P*=0.0116, *****P*<0.0001), and TNF-α (*****P*<0.0001) after 6h or 24h of 100 ng/mL LPS stimulation compared to untreated cells. WT vs M114T$^{+/-}$ levels of IL-6 at 6h LPS ($^{\#}P$ = 0.0444) and WT vs M114T+/+ levels of IL-10 at 0h ($^{\#}P$=0.0234) were significantly different. All other WT vs M114T$^{+/-}$ and WT vs M114T$^{+/+}$ comparisons were not statistically significant. Statistics were determined by two-way ANOVA and Šídák's multiple comparisons test. P-values are listed only for WT cells for simplicity; all other statistical comparisons are defined in **Data S1**. Mean ± SEM for n=3-4 independent differentiations is shown for all bar graphs, with each data point representing an individual differentiation. Source data are provided as a Source Data file.

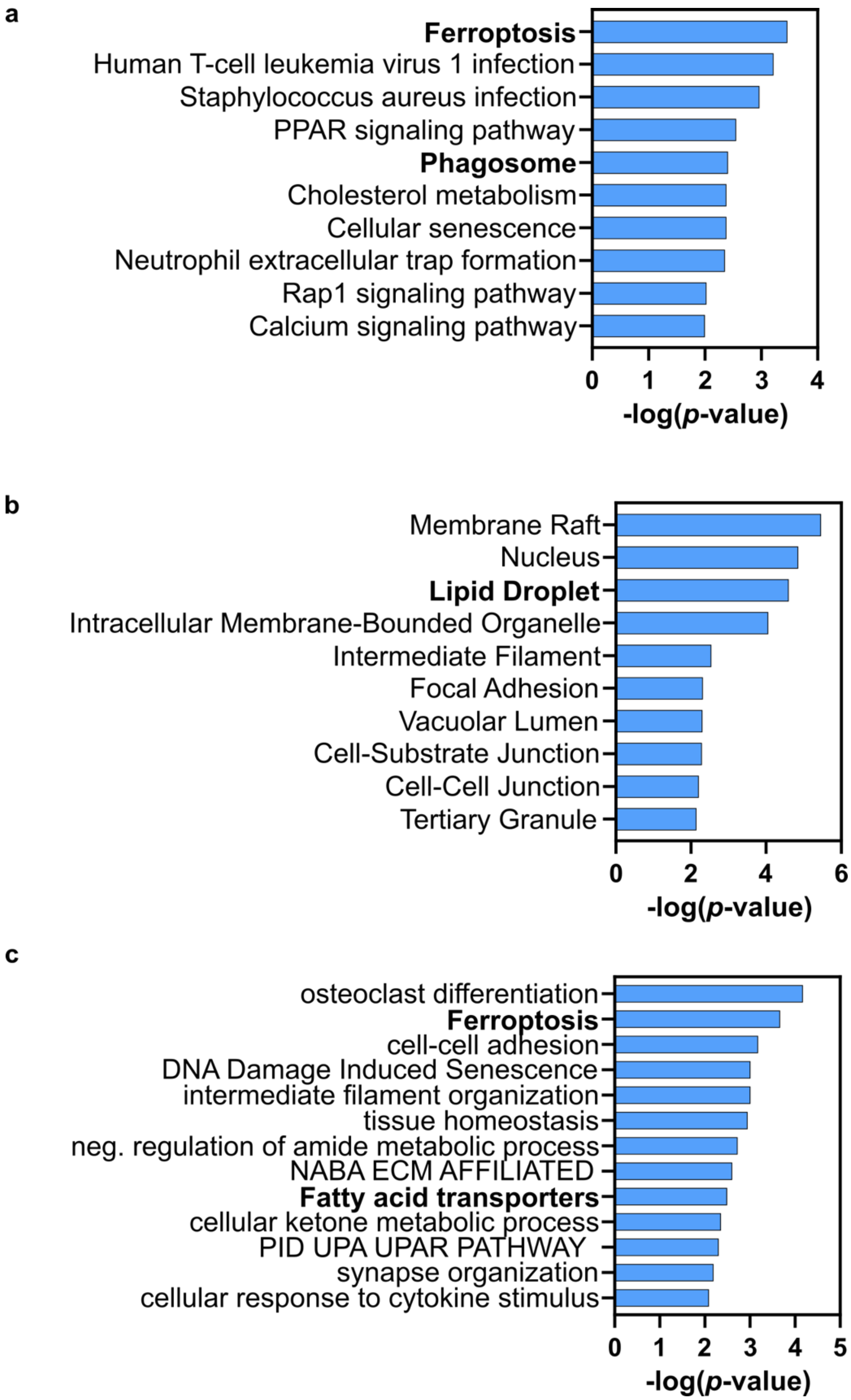

Supplemental Figure 4. Additional functional enrichment analyses of differentially expressed proteins from the proteomics analysis of C71G$^{+/-}$ versus WT iMGs. a,b Enriched terms obtained by Enrichr for KEEG pathway (**a**) and gene ontology cellular component (**b**) libraries. P-values were computed using a two-sided Fischer's exact test. **c,** Enriched terms generated by Metascape analysis. P-values were determined using the hypergeometric test and

Benjamini-Hochberg algorithm. Top 10 terms are shown for each analysis. Additional terms and statistical values can be found in **Data S4**. Source data are provided as a Source Data file.

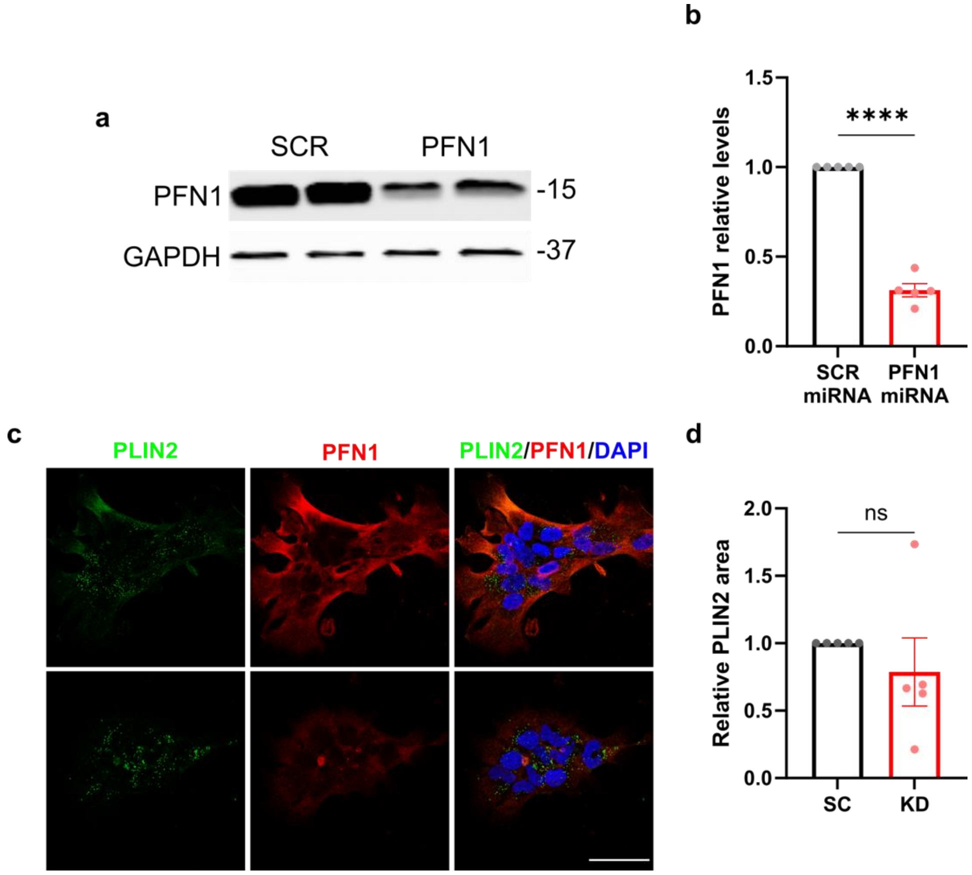

Supplemental Figure 5. PFN1 knockdown does not correlate with an increase in lipid droplet accumulation in HMC3 microglial cells. PFN1 knockdown in HMC3 cells using miRNAs targeting *PFN1* or a scrambled (SCR) sequence for n=5 independent experiments. **a,** Representative Western blot of PFN1 and GAPDH used as loading control. **b,** Quantification of PFN1 (****P<0.0001, t = 18.88, df = 8) levels from Western blots exemplified in **a**. PFN1 levels were normalized to GAPDH and to SCR controls for each independent experiment. **c-d** Immunofluorescence analysis of lipid droplet accumulation in HMC3 cells with SCR or PFN1 miRNA. **c,** Representative immunofluorescent images of PLIN2 and PFN1. Scale bar: 50µm. **d,** Quantification of the area of PLIN2 fluorescent punctate signal representing lipid droplets was normalized to SCR miRNA HMC3s within each independent experiment (ns P=0.4231, t=0.8442, df=8). All graphs show mean ± SEM. Data points in bar graphs represent individual experiments. Statistics were performed by unpaired two-tailed t-test. Source data are provided as a Source Data file.

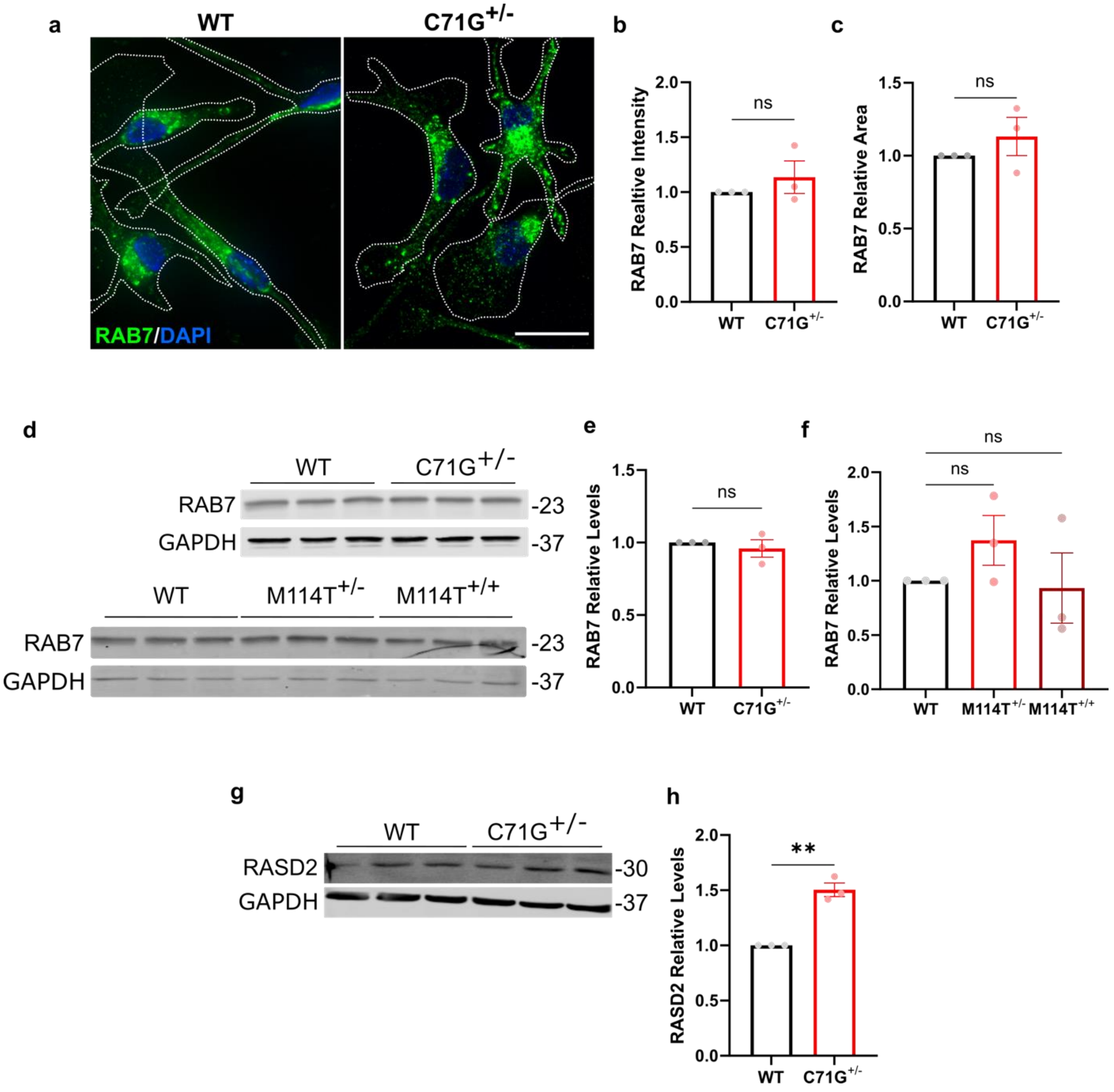

Supplemental Figure 6. Additional validations related to RNASeq analyses of PFN1 iMGs. **a-c,** Immunofluorescence analysis of RAB7 in WT and PFN1 C71G$^{+/-}$ iMGs (n=3 independent differentiations). **a,** Representative immunofluorescence images. Cell boundaries defined by anti-PFN1 staining (not shown for clarity) are depicted with white dashed lines. Scale bar=50 µm. **b,c,** Quantification of the mean intensity (**b,** ns *P*=0.4094, t=0.9205, df=4) and area (**c,** ns *P*= 0.3701, t=1.009, df=4) of RAB7 immunofluorescence signal analyzed on a per cell basis. Mutant iMG data were normalized to the corresponding WT condition within the same experimental differentiation.

d, Western blot analysis of RAB7 with GAPDH used as a loading control for cell lysates derived from C71G$^{+/-}$, M114T$^{+/-}$, M114T$^{+/+}$ iMGs and their respective WT iMG counterpart (n=3 independent differentiations). **e**, Quantification of **d** for WT and C71G$^{+/-}$ iMGs (ns *P*=0.5405, t=0.6683, df=4). **f,** Quantification of **d** for WT, M114T$^{+/-}$ and M114T$^{+/+}$ iMGs (ns *P*=0.2518, q= 1.633, df=6 for WT vs M114T$^{+/-}$ and ns *P*= 0.7486, q= 0.6546, df=6 for WT vs M114T$^{+/+}$). For each independent differentiation, RAB7 protein levels were normalized to the levels of the respective WT controls from the same experimental differentiation. **g,** Western blot analysis of RASD2 including GAPDH loading control for cell lysates derived from C71G$^{+/-}$ and WT iMGs (n=3 independent differentiations). **h,** Quantification of **g** (***P*=0.0012, t=8.148, df=4). For each independent differentiation, RASD2 protein levels were normalized to the levels of the respective WT controls from the same experimental differentiation. All bar graphs show mean ± SEM with individual data points representing independent differentiations. Statistics were determined using unpaired two-tailed t-test for WT vs C71G$^{+/-}$ iMGs comparisons and ordinary one-way ANOVA with Dunnett's multiple comparisons test for WT vs M114T$^{+/-}$ and M114T$^{+/+}$ comparisons. Source data are provided as a Source Data file.

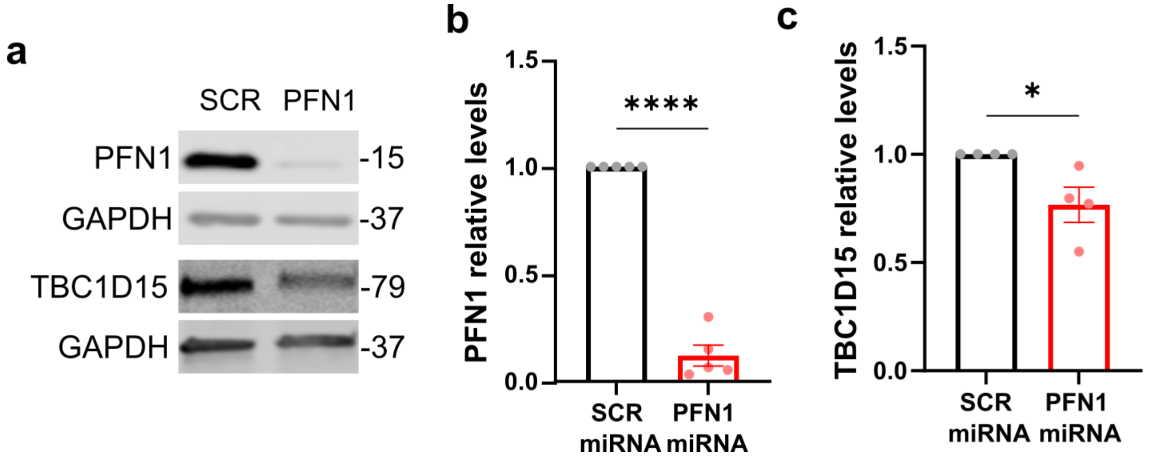

Supplemental Figure 7. PFN1 knockdown in HMC3 microglia cells correlates with reduced TBC1D15 levels. PFN1 knockdown in HMC3 cells using miRNAs targeting *PFN1* or a scrambled (SCR) sequence for n=5 independent experiments. **a,** Representative Western blot of PFN1, TBC1D15 and GAPDH, used as loading control. **b,** Quantification of PFN1 (*****P*=<0.0001, t=17.82, df=8) levels from Western blots exemplified in **a**. **c,** Quantification of TBC1D15 (**P*=0.0289, t=2.857, df=6) as described in **b**. **b,c,** The indicated protein was normalized to GAPDH and to SCR controls for each independent experiment. All graphs show mean ± SEM. Data points in bar graphs represent individual experiments. Statistics were performed by unpaired two-tailed t-test. Source data are provided as a Source Data file.

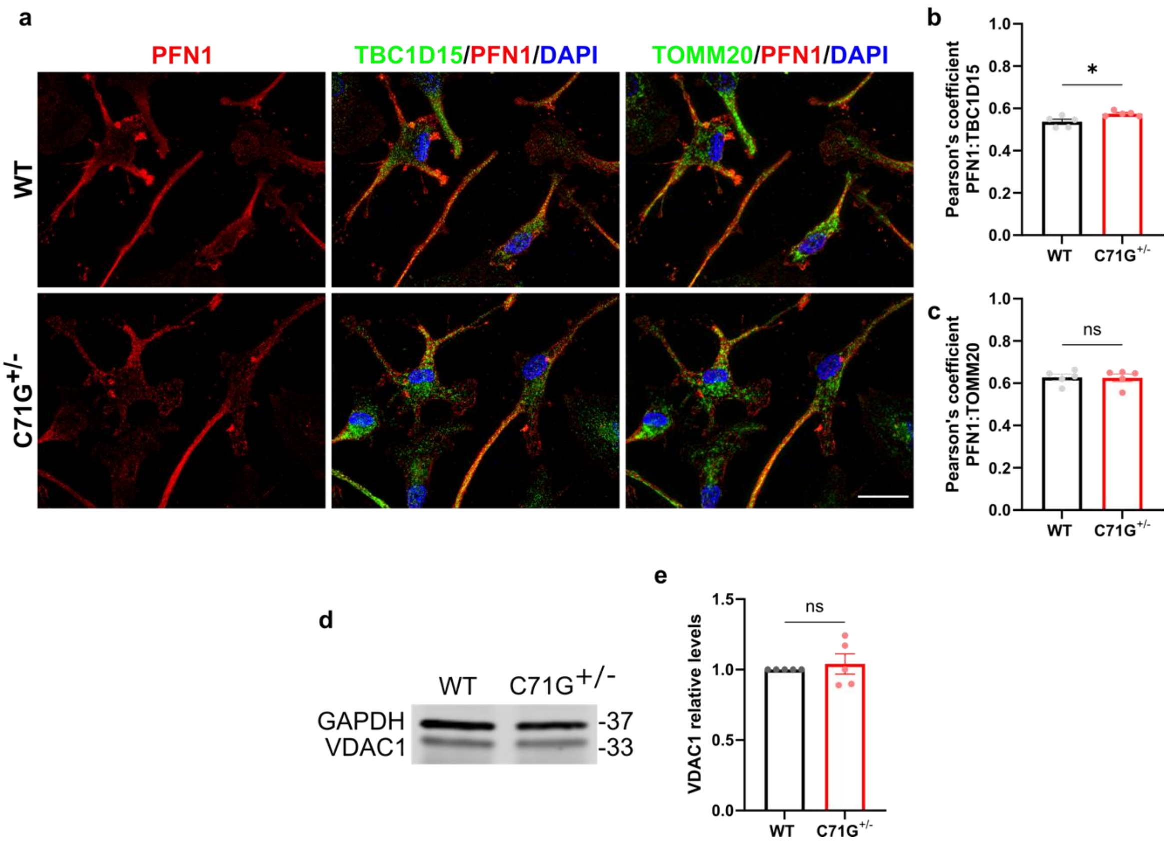

Supplemental Figure 8. Colocalization analysis of PFN1 with TBC1D15 and TOMM20 and mitochondrial levels in PFN1 WT and C71G$^{+/-}$ iMGs. a-c, Colocalization of PFN1 with TBC1D15 and TOMM20 signal as determined by immunofluorescence analysis from n=4 independent differentiations. **a,** Representative immunofluorescence images of PFN1, TBC1D15, and TOMM20 staining in PFN1 WT and C71G$^{+/-}$ iMGs. **b,c,** Pearson's correlation coefficient of PFN1 and TBC1D15 signal (**b,*** *P* = 0.0181, t=2.961,df=8) and PFN1 and TOMM20 signal (**c,** ns *P*=0.9261,t=0.09571, df=8). **d,** VDAC1 protein expression in PFN1 WT and C71G$^{+/-}$ iMGs determined by Western blot analysis. **e,** Quantification of **d.** VDAC1 levels were normalized to GAPDH and then to the levels of the respective WT line from the same differentiation (ns *P*=0.5898, t= 0.5615, df=8) for n=5 independent differentiations. All bar graphs show mean ± SEM where each data point represents an independent differentiation. Statistics were determined using an unpaired two-tailed t-test. Source data are provided as a Source Data file.

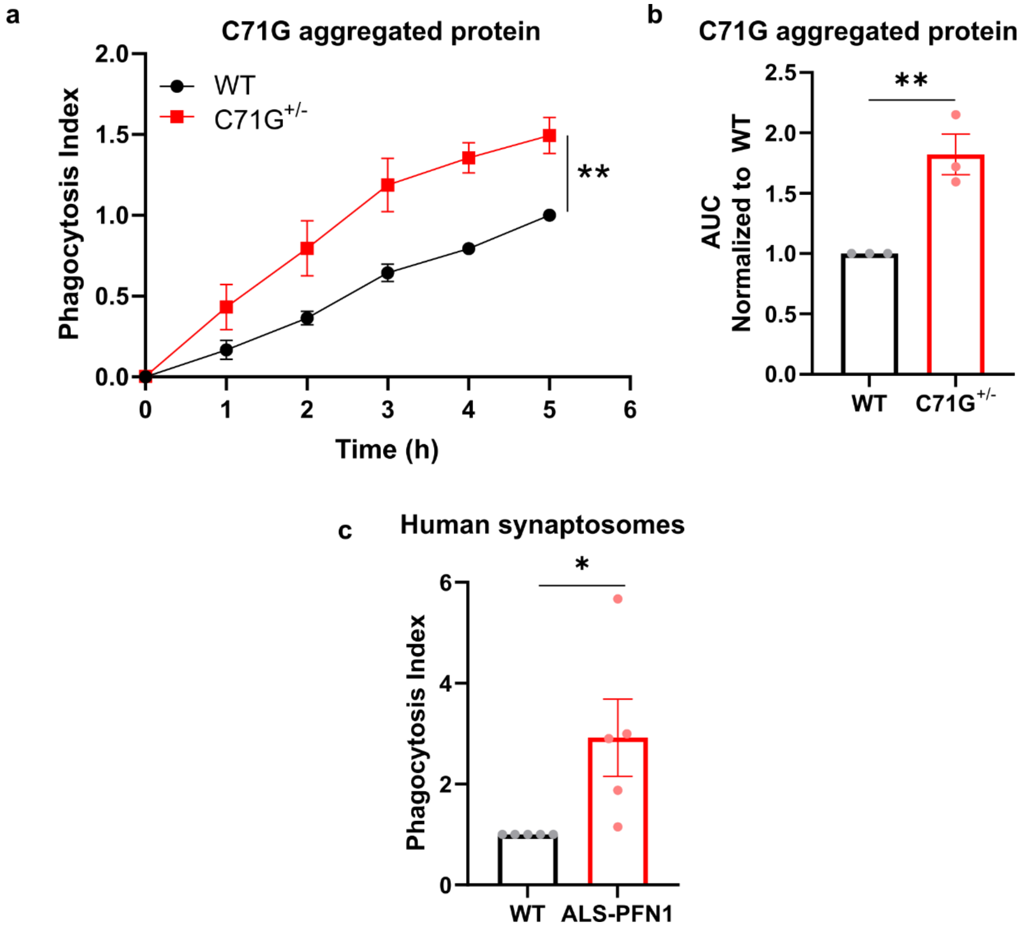

Supplemental Figure 9. Differential phagocytosis indices for ALS-PFN1 and WT iMGs upon administration of disease-relevant substrates. a-c, Live-cell phagocytosis assays using pHrodo-labeled disease-relevant substrates. **a,** Quantification of the phagocytosis index (see Methods) for WT and C71G$^{+/-}$ iMGs (n=3 independent differentiations) in the presence of pHrodo-labeled PFN1 C71G aggregated purified protein over a time course of 5h (paired two-tailed t-test, ***P*= 0.0072, t=4.376, df=5). **b,** Area under the curve (AUC) determined from **a** (unpaired two-tailed t-test, ***P*= 0.0082, t=4.881, df=4). **c**, Quantification of the phagocytosis index for C71G$^{+/-}$ (n=3 independent differentiations) and M114T$^{+/-}$ (n=2 independent differentiations), referred to collectively as "ALS-PFN1", and their respective WT controls (n=5 independent differentiations) in the presence of human synaptosomes after 2h of phagocytosis (unpaired two-tailed t-test, **P*= 0.0369, t=2.501, df=8). Mutant iMG data was normalized to the levels of the respective WT control from the same experimental differentiation. All graphs show mean ± SEM with individual data points representing independent differentiations. Source data are provided as a Source Data file.

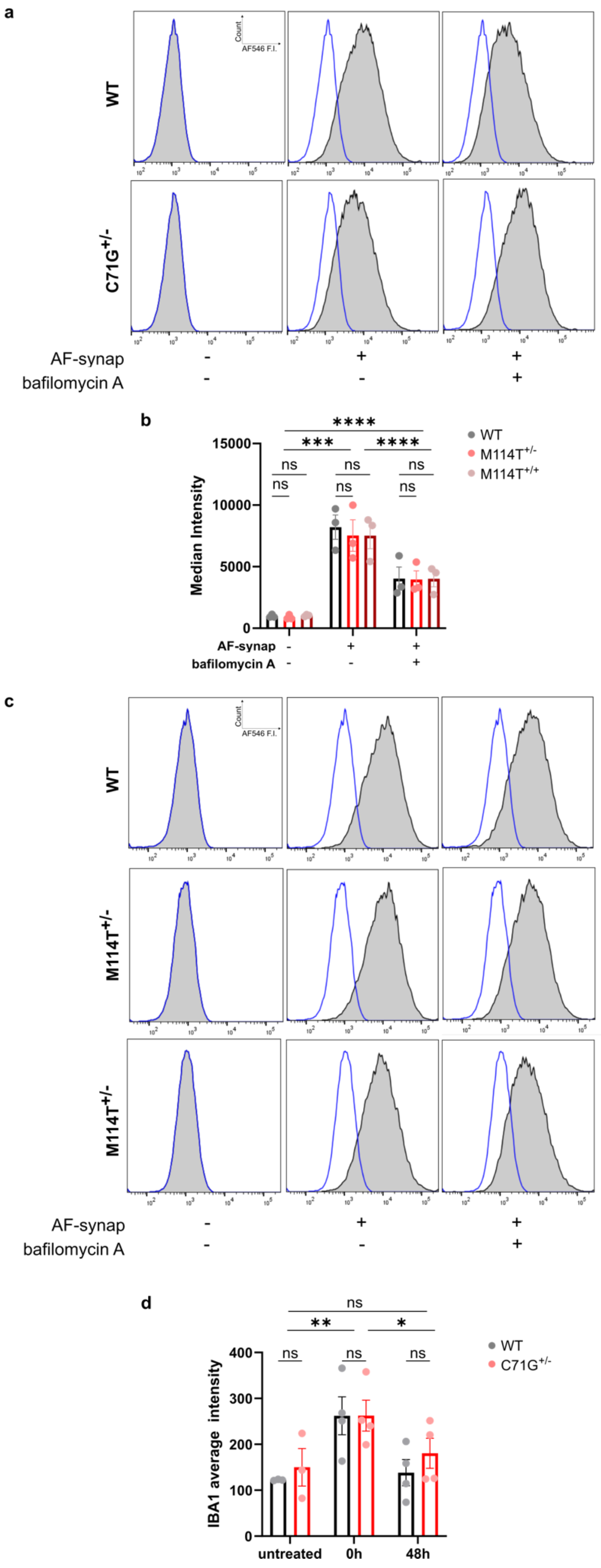
a
WT
C71G+/-
Count
AF546 F.I.
AF-synap - + +
bafilomycin A - - +
b
Median Intensity
15000
10000
5000
0
****
***
****
ns
ns
WT
M114T+/-
M114T+/+
AF-synap - + +
bafilomycin A - - +
c
WT
M114T+/-
M114T+/-
Count
AF546 F.I.
AF-synap - + +
bafilomycin A - - +
d
IBA1 average intensity
400
300
200
100
0
ns
**
*
WT
C71G+/-
untreated
0h
48h

Supplemental Figure 10. Analysis of synaptosome uptake and IBA1 levels across the washout assay. a-c, Synaptosome uptake measured by flow cytometry in PFN1 WT and C71G$^{+/-}$ (and PFN1 WT, M114T$^{+/-}$, and M114T$^{+/+}$ iMGs (n=3 independent differentiations for each line). **a,** Representative fluorescence histograms from flow cytometry depicting uptake of AF546-labeled synaptosomes (AF-synap) by WT and C71G$^{+/-}$ iMGs. AF-synap intensity is shown in the X-axis. Histograms of iMGs without synaptosomes (shown in blue without shading) are compared to histograms of iMGs incubated with synaptosomes with or without pre-treatment with BafA (shown in black with shading). **b,** Quantification of flow cytometry assay measuring uptake of AF-synap in WT, M114T$^{+/-}$, and M114T$^{+/+}$ iMGs. No WT vs M114T$^{+/-}$ or WT vs M114T$^{+/+}$ comparisons were statistically significant (two-way ANOVA with Šídák's multiple comparisons test). iMGs pre-treated with BafA showed an attenuation but not removal of uptaken AF-synap (*****P*<0.0001, t=5.846, df = 18 for +BafA vs -BafA). Additional statistical comparisons are in **Data S1**. Data points in the graph represent independent differentiations. **c,** Representative fluorescence histograms from flow cytometry for WT, M114T$^{+/-}$, and M114T$^{+/+}$ iMGs as described in **a**. **d,** Quantification of IBA1 mean intensity before addition of synaptosomes (untreated), 0h and 48h post-synaptosome washout as indicated in **Figure 4f**. No WT vs C71G$^{+/-}$ comparisons were statistically significant (two-way ANOVA with Šídák's multiple comparisons test; ns *P*= 0.9386, t= 0.5268 for untreated; ns *P*= >0.9999, t= 0.006518 for 0h and ns *P*= 0.7478 t= 0.9261 for 48h; df=16 for all groups). IBA1 intensity is increased at 0h post-washout and returns to initial levels at 48h post-washout (two-way ANOVA with Tukey's multiple comparison test; ** *P*=0.0062, q= 5.110, df=16; * *P*= 0.0150, q= 4.504, df=16; ns P=0.7867, q= 0.9404, df=16). Immunofluorescence signal was analyzed on a per cell basis for n=3-4 independent differentiations. All bar graphs show mean ± SEM. Source data are provided as a Source Data file.

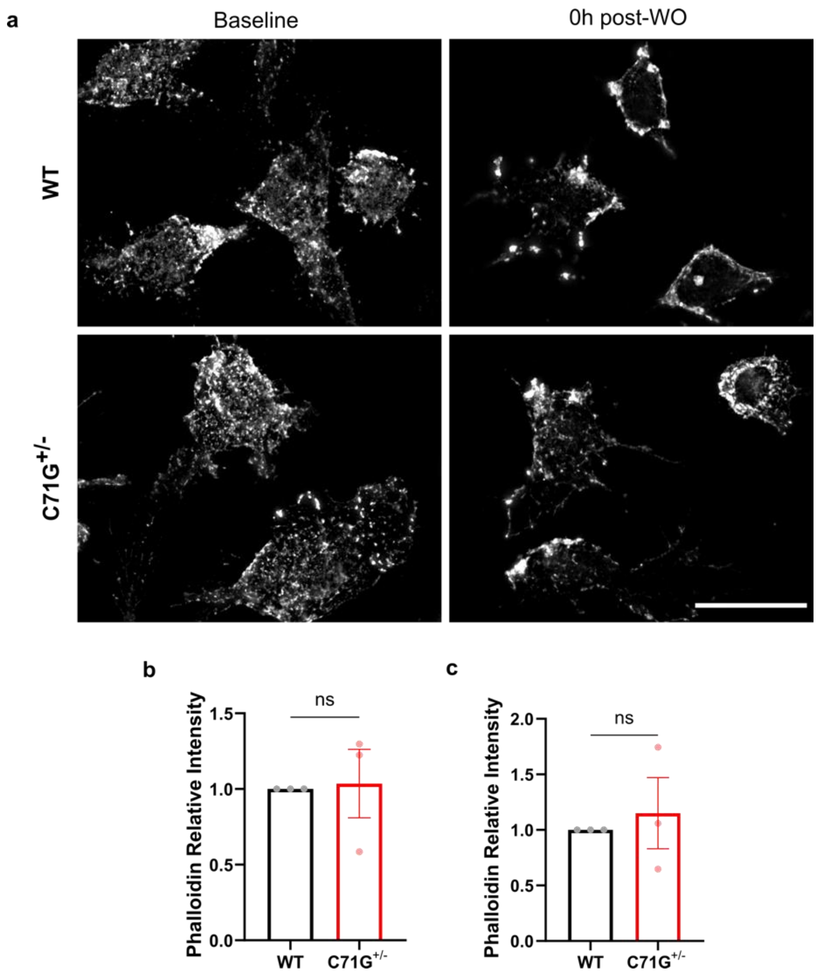

Supplemental Figure 11. Analysis of F-actin detected by phalloidin staining of PFN1 C71G$^{+/-}$ and WT iMGs subjected to the live-cell phagocytosis assay. a, Representative immunofluorescence images of phalloidin staining of PFN1 WT and C71G$^{+/-}$ iMGs before administration of human synaptosomes (baseline) and immediately after synaptosomes were washed out (0h post-WO). Scale bar: 25 µm **b,c,** Quantification of phalloidin mean fluorescent intensity for baseline (**b**) and 0h post-WO (**c**). Data from C71G$^{+/-}$ iMGs was normalized to that of WT iMGs within the same experimental differentiation. All graphs show mean ± SEM with individual data points representing n=3 independent differentiations. Unpaired two-tailed t-test was used for all statistical comparisons (**b,** ns P=0.8882, t=0.1591, df=2 and **c,** ns P=0.6621, t=0.4711, df=4). Source data are provided as a Source Data file.

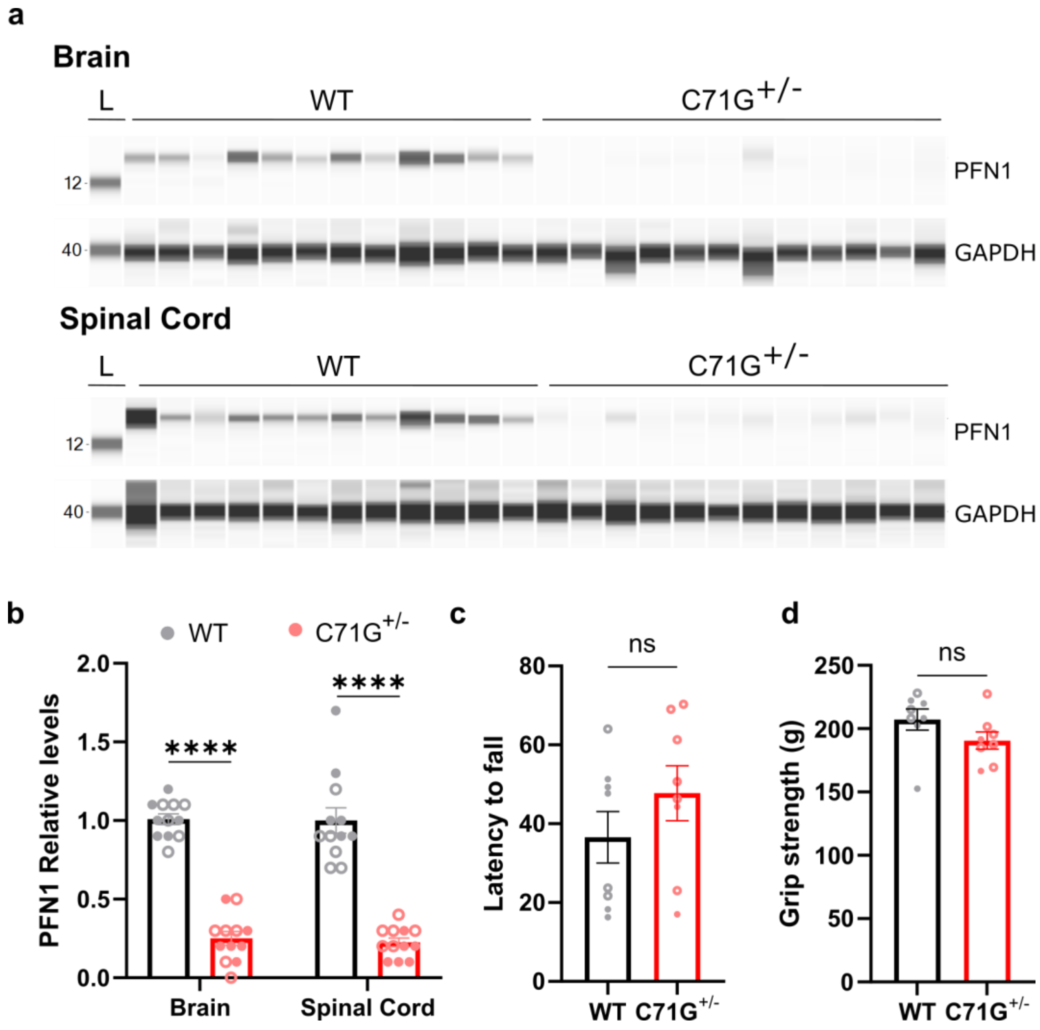

Supplemental Figure 12. Characterization of ALS-PFN1 mice. a, Capillary Western lane view image showing a ladder (L) as well as PFN1 and GAPDH (loading control) levels in brain and spinal cord lysates from n = 12 WT control (6 male, 6 female) and 12 PFN1 C71G$^{+/-}$ (6 male, 6 female) mice. **b,** Quantification of PFN1 levels in brain (*****P*= <0.0001, t = 10.48, df = 44) and spinal cords (*****P*= <0.0001, t = 10.71, df = 44) from **a**. Two-way ANOVA with Šídák's multiple comparisons test was used for statistical comparisons. **c-d** Motor function assessment of WT and PFN1 C71G$^{+/-}$ animals. Two-tailed unpaired t-test was used for statistical comparisons. **c,** Latency to fall in a Rotarod test (ns *P*=0.2604, t=1.173, df = 14). **d,** Grip strength of all four limbs (ns *P*=0.1450, t=1.544, df=14). Two-tailed unpaired t test was used for statistical comparisons in **c,d**. Bar graphs show mean ± SEM. Data points represent individual animals. Open symbols for males and closed symbols for females. Source data are provided as a Source Data file.

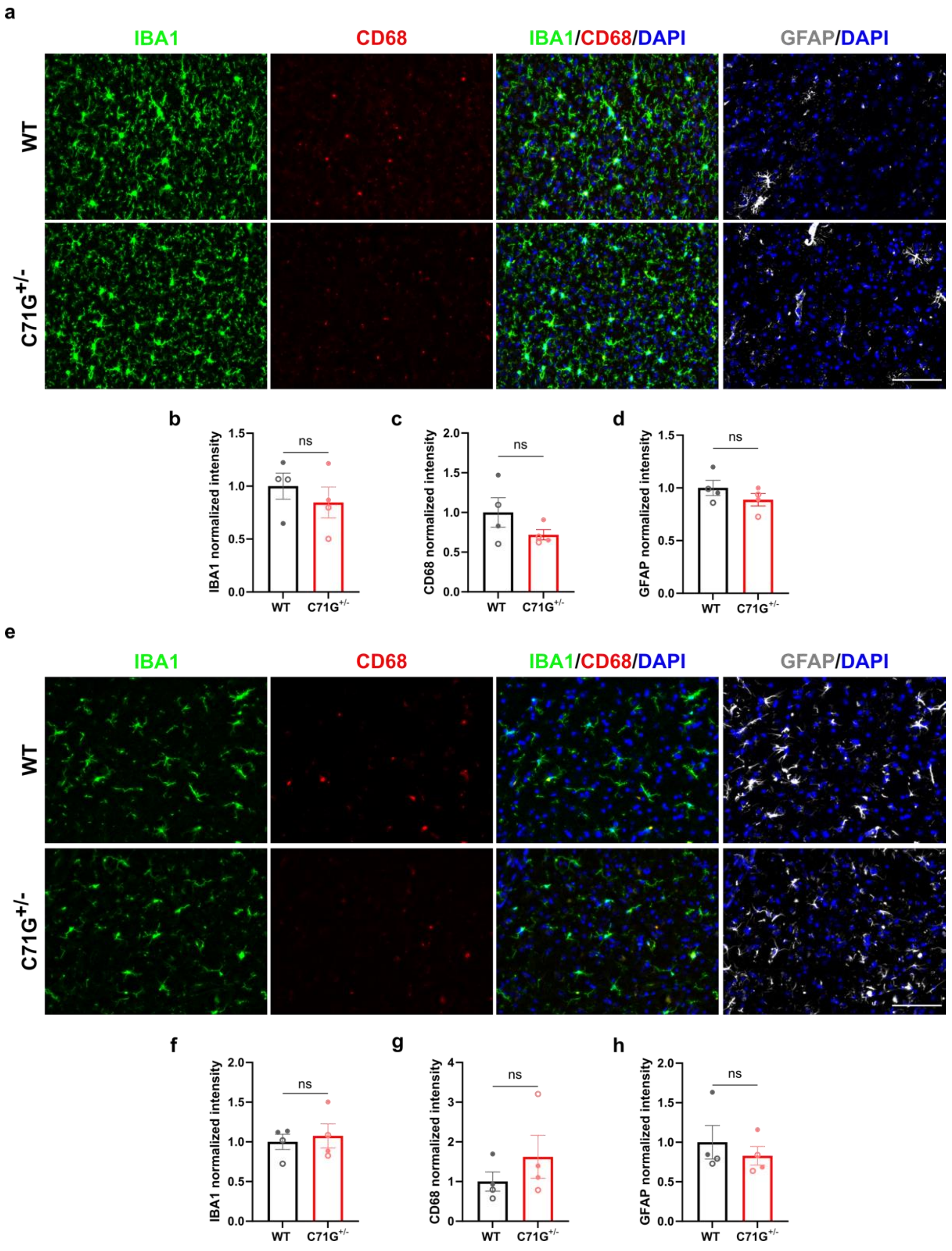

Supplemental Figure 13. Glial marker expression is similar between aged WT and ALS-PFN1 mice. a, Representative immunofluorescence images of IBA1 (green), CD68 (red) and GFAP (grey) staining in motor cortex of WT and PFN1 C71G$^{+/-}$ mice. Note that GFAP images were acquired separately from Iba1/CD68. Scale bar: 100 µm. **b-d,** Quantification of the normalized intensity with respect to the average of the total signal intensity in the WT mice for IBA1 (**b,** ns P=0.4521, t=0.804, df=6), CD68 **(c,** ns P=0.2037, t=1.426, df=6), and GFAP (**d,** ns P=0.2714, t=1.211, df=6). **e,** Representative immunofluorescence images of spinal cord as

described in **a**. Scale bar: 100 µm. **f-h**, Quantification of normalized intensity as mentioned above for IBA1 (**f,** ns *P*=0.6918, t=0.416, df=6), CD68 (**g,** *P*=0.3349, t=1.048, df=6) and GFAP (**h,** ns *P*=0.5103, t=0.700, df=6). Unpaired two-tailed t-test was used for all statistical comparisons. Graphs in this figure show mean ± SEM from n=4 WT (2 male, 2 female) and 4 C71G$^{+/-}$ (2 male, 2 female) mice. Data points represent individual animals, with open symbols for males and closed for female. Source data are provided as a Source Data file.

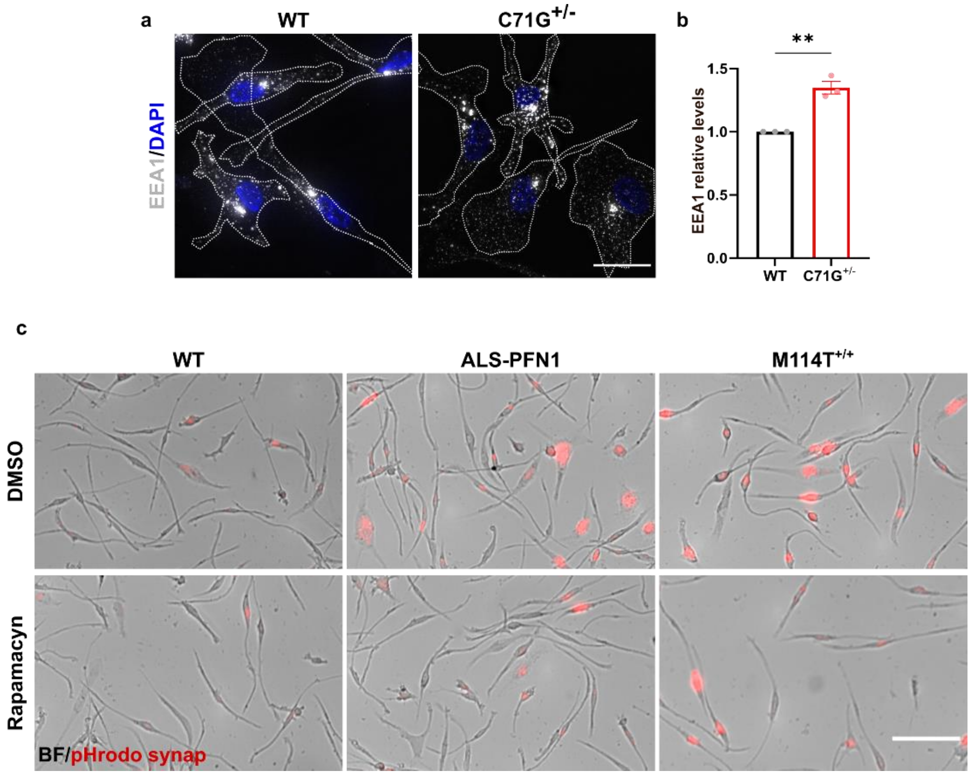

Supplemental Figure 14. EEA1 levels at baseline and live-cell phagocytosis assay after rapamycin treatment in PFN1 C71G$^{+/-}$ and WT iMGs. a, Representative immunofluorescence images of EEA1 (n=3 independent differentiations) for PFN1 WT and C71G$^{+/-}$ iMGs. **b,** Quantification of the EEA1 signal intensity measured per cell and normalized to WT iMGs (***P*=0.0021, t=7.070, df=4). Data points represent independent differentiations. **c,** Representative live-cell images of phagocytosis assay after 8h of incubation with pHrodo-labeled mouse synaptosomes (pHrodo synap) treated with DMSO or 0.1µM rapamycin for WT, M114T$^{+/-}$ (ALS-PFN1) and M114T$^{+/+}$ iMGs. Cellular morphology is depicted by bright field (BF) images merged with pHrodo synap signal in red. Scale bar= 25µm. Source data are provided as a Source Data file.

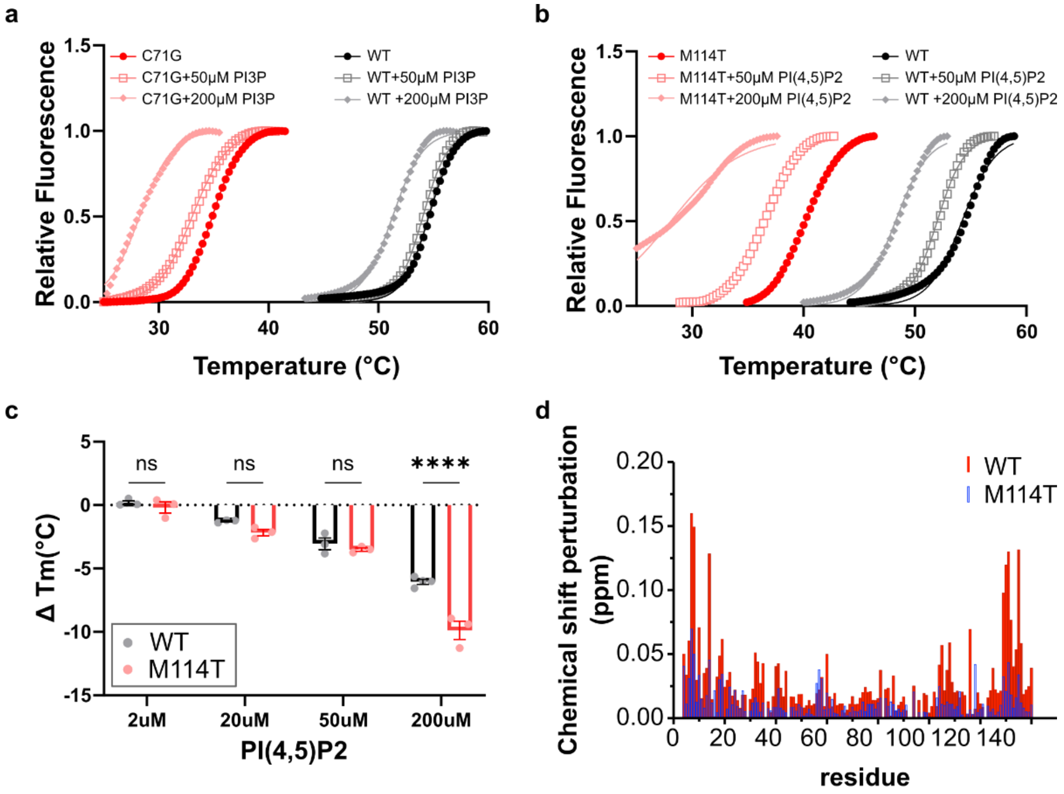

e

SSE	Residues
α_1	4-12
β_1	17-24
β_2	30-34
3_{10}	38-41
α_2	45-52
α_3	56-62
β_3	64-66
β_4	69-77
β_5	85-90
β_6	99-105
β_7	109-115
α_4	121-138

Supplemental Figure 15. Additional data pertaining to binding of purified PFN1 variants with phosphoinositides. a,b, Thermal denaturation profiles of PFN1 proteins incubated with different concentrations of PI3P measured by SYPRO Orange fluorescence as a function of increasing temperature as described for main text **Figure** 8 for PFN1 C71G (**a**) and M114T (**b**) . An average of two technical replicates is shown, which are representative of n=3-4 independent experiments for **b**. The curves were fit with the Boltzmann's sigmoidal function to determine an apparent melting temperature (T_m). **c**, ΔT_m reflects the difference between the T_m of the PFN1 M114T variant with the indicated concentration of PI(4,5)2P and the T_m of that PFN1 variant without PI(4,5)2P. Statistics were determined using two-way ANOVA F (1, 28) = 7.115 and Šídák's multiple comparisons test (*****P*=<0.0001 and ns *P*=0.2851, 0.7847). Bar graph shows mean ±

SEM with each data point representing an independent experiment. **d,e,** Additional information for the NMR titration studies of PFN1 with PI3P as described in main text **Figure 8 d,** Chemical shift perturbation (ppm) for each residue of PFN1 between the free and the PI3P-bound state for PFN1 WT (red lines) and PFN1 M114T (blue lines). The secondary structural elements (SSE) formed by the indicated PFN1 sequences are shown and defined as per[1]. Source data are provided as a Source Data file.

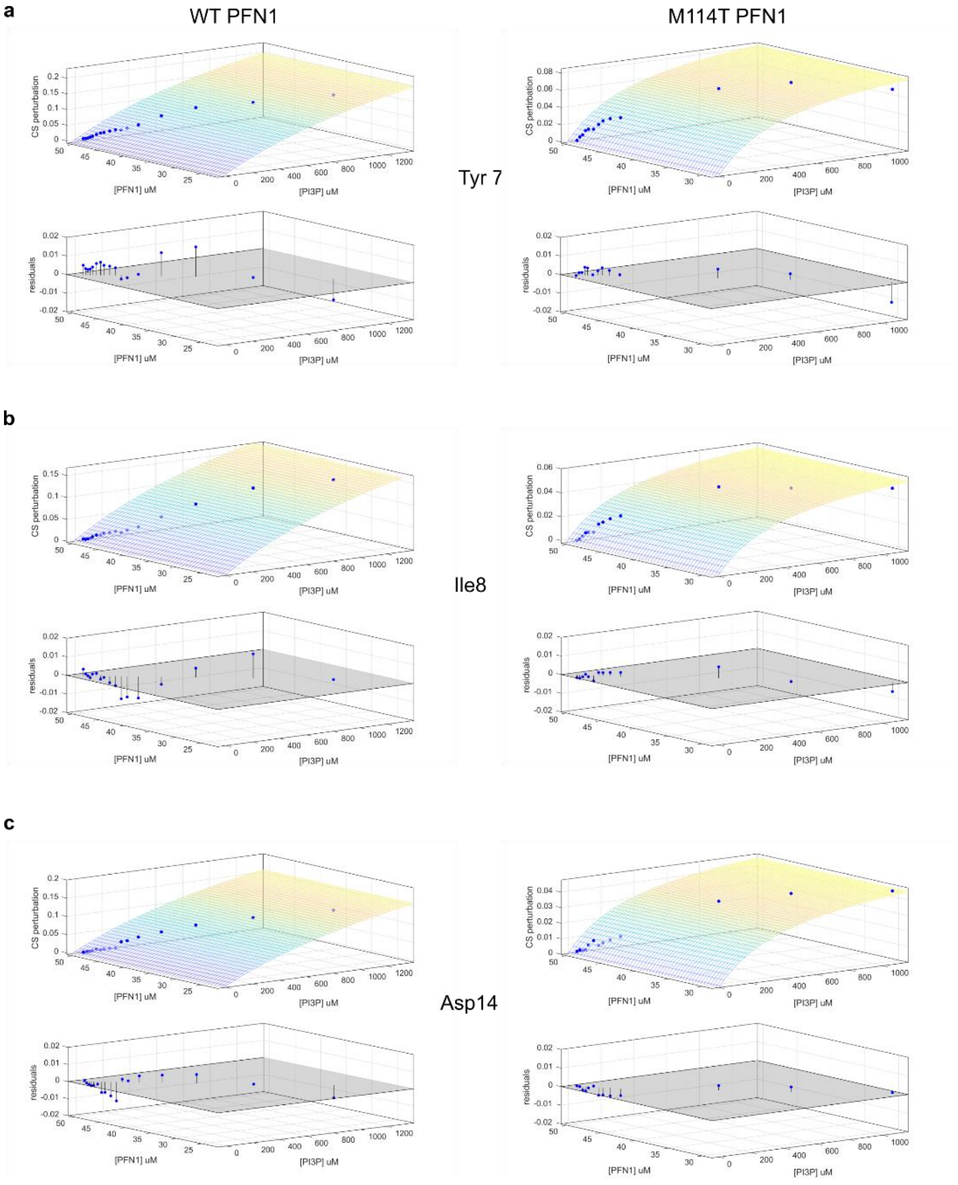

Supplemental Figure 16. Representative residuals of the MATLAB fits for the NMR titration data for PFN1 with PI3P. The change in chemical shift (CS perturbation in ppm) for the indicated PFN1 residue (Tyr 7, Ile8 and Asp14) is plotted as a function of PFN1 concentration (uM) and PI3P concentration for both PFN1 WT (left column) and PFN1 M114T (right column). The resultant titration curves were fitted in MATLAB to obtain a dissociation constant as described in the methods. Residuals of the fitting are shown in blue. Source data are provided as a Source Data file.

SUPPLEMENTARY REFERENCES

1. Schmidt, E.J. et al. ALS-linked PFN1 variants exhibit loss and gain of functions in the context of formin-induced actin polymerization. *Proceedings of the National Academy of Sciences* **118**(2021).
