# Supplementary material for: Expression of ALS-PFN1 impairs vesicular degradation in iPSC-derived microglia
Source: Nat Commun. 2024 Mar 20;15:2497. doi: 10.1038/s41467-024-46695-w (PMC10954694; doi:10.1038/s41467-024-46695-w)
Supplement: Supplementary file 3 — Description of Additional Supplementary Files [file 41467_2024_46695_MOESM3_ESM.pdf]

Description of Additional Supplementary Files

Data S1. Additional statistical analysis for this study. The file contains results for statistical analysis in: **Figure 1c**, statistics for C71G$^{+/-}$ cells (iMGs vs PMPs and iPSCs) using two-way ANOVA and Šídák's multiple comparisons test. **Figure 1e**, additional comparisons not listed in the main text figure for WT iMGs and all comparisons for PFN1 C71G$^{+/-}$ iMGs, including LPS treated versus untreated conditions, using two-way ANOVA and Šídák's multiple comparisons test. **Sup. Figure 3,** additional comparisons not listed in Supplementary Figure 3 for WT iMGs and all comparisons for PFN1 M114T$^{+/-}$ and M114T$^{+/+}$ iMGs, including LPS treated versus untreated conditions, using two-way ANOVA and Šídák's multiple comparisons test. **Figure 4b and d**, the indicated comparison was assessed by two-way ANOVA followed by Tukey's multiple comparisons for WT vs C71G$^{+/-}$ iMGs (**b**) and WT vs M114T$^{+/-}$ vs M114T$^{+/-}$ iMGs (**d**). **Figure 4i**, statistics for WT vs C71G$^{+/-}$ iMGs within each treatment condition and additional comparisons not listed in main text figure across different treatment conditions, using two-way ANOVA and Šídák's multiple comparisons test. **Sup. Figure 7**, Additional comparisons not listed in Supplementary Figure 7 as in Figure 4i. **Figure 7e**, Additional comparisons not listed in the main text figure for WT iMGs untreated vs rapamycin and C71G$^{+/-}$ iMGs for both untreated vs rapamycin and untreated vs bafilomycin A.

Data S2. Results of the differential expression analysis of C71G$^{+/-}$ vs WT iMGs using quantitative proteomics. Data resulting from the mass spectrometry experiment using tandem mass tag (TMT) quantitative proteomics to identify differentially expressed proteins between PFN1 WT and C71G$^{+/-}$ iMGs. PFN1 WT iMGs was used as the reference condition. Data was analyzed using Scaffold Software as described in the methods. Statistics were determined using T-test and Benjamin-Hochberg correction for multiple testing. Differentially expressed proteins in herein have a *P*- value<0.00160 and are considered statistically significant with the Benjamin-Hochberg test.

Data S3. Functional enrichment analysis of differentially expressed proteins identified from the quantitative proteomics study. Enriched pathways identified using the Bioplanet 2019 library by Enrichr. Differentially expressed proteins used for this analysis are listed in **Data S2**.

Data S4. Enrichr and Metascape analyses of differentially expressed proteins from the quantitative proteomics study. Enrichr (KEEG pathway and Gene Ontology celullar component) and Metascape were used to determine enriched functional terms from the differentially expressed proteins listed in **Data S2**. The results of each analysis are presented in different tabs.

Data S5. Results of differential gene expression analysis from the RNASeq dataset. RNAseq data obtained from C71G$^{+/-}$ (CG) and M114T$^{+/-}$ (MThet) iMGs (ALS-PFN1 group) and WT controls (WT and WT2) were analyzed using DESeq2 package and Wald test.

Data S6. Information for antibodies used herein. The target, antibody species, clone information, supplier, catalog number (#), lot number, application and working dilution are provided.

Supplementary Code 1. ImageJ analysis script used to measure Iba1 signal in dead neuron injections tissue.
