# Supplementary material for: Expression of ALS-PFN1 impairs vesicular degradation in iPSC-derived microglia
Source: Nat Commun. 2024 Mar 20;15:2497. doi: 10.1038/s41467-024-46695-w (PMC10954694; doi:10.1038/s41467-024-46695-w)
Supplement: Supplementary file 4 — Data S1 [file 41467_2024_46695_MOESM4_ESM.docx]

| **Figure 1c** | | | | | |
| --- | --- | --- | --- | --- | --- |
| **Gene** | **Comparison for C71G^+/-^ cells** | | ***P value*** | | |
| *PROS1* | iPSCs vs. iMGs | | 0.0032 | | |
| *PROS1* | PMPs vs. iMGs | | 0.0212 | | |
| *GPR34* | iPSCs vs. iMGs | | 0.0035 | | |
| *GPR34* | PMPs vs. iMGs | | 0.0124 | | |
| *P2RY12* | iPSCs vs. iMGs | | 0.0019 | | |
| *P2RY12* | PMPs vs. iMGs | | 0.0019 | | |
| *MERTK* | iPSCs vs. iMGs | | 0.0013 | | |
| *MERTK* | PMPs vs. iMGs | | 0.0047 | | |
| *SPI-1* | iPSCs vs. PMPs | | 0.0209 | | |
| *SOX2* | iPSCs vs. iMGs | | <0.0001 | | |
| **Figure 1e** | | | | | |
| **Cytokine** | **Other comparison for WT iMGs** | | ***P value*** | | |
| IL-6 | Untreated vs 6h LPS | | 0.3593 | | |
| IL-6 | 6h LPS vs 24h LPS | | 0.09 | | |
| IL-10 | Untreated vs 6h LPS | | 0.3375 | | |
| IL-10 | 6h LPS vs 24h LPS | | 0.2764 | | |
| CCL5 | Untreated vs 6h LPS | | 0.9907 | | |
| CCL5 | 6h LPS vs 24h LPS | | 0.0127 | | |
| TNF-α | Untreated vs 24h LPS | | 0.9964 | | |
| TNF-α | 6h LPS vs 24h LPS | | 0.03 | | |
| **Cytokine** | **Comparison for C71G^+/-^ iMGs** | | ***P value*** | | |
| IL-6 | Untreated vs 6h LPS | | 0.4469 | | |
| IL-6 | Untreated vs 24h LPS | | 0.0146 | | |
| IL-6 | 6h LPS vs 24h LPS | | 0.1873 | | |
| IL-10 | Untreated vs 6h LPS | | 0.2287 | | |
| IL-10 | Untreated vs 24h LPS | | 0.0018 | | |
| IL-10 | 6h LPS vs 24h LPS | | 0.0543 | | |
| CCL5 | Untreated vs 6h LPS | | 0.9832 | | |
| CCL5 | Untreated vs 24h LPS | | 0.0076 | | |
| CCL5 | 6h LPS vs 24h LPS | | 0.0141 | | |
| TNF-α | Untreated vs 6h LPS | | 0.0125 | | |
| TNF-α | Untreated vs 24h LPS | | 0.9967 | | |
| TNF-α | 6h LPS vs 24h LPS | | 0.0177 | | |
| **Cytokine** | **Comparisons for Untreated iMGs** | | ***P value*** | | |
| IL-6 | WT vs C71G^+/-^ | | >0.9999 | | |
| IL-10 | WT vs C71G^+/-^ | | 0.9257 | | |
| CCL5 | WT vs C71G^+/-^ | | 0.9987 | | |
| TNF-α | WT vs C71G^+/-^ | | 0.8306 | | |
| **Cytokine** | **Other comparison for 6h LPS iMGs** | | ***P value*** | | |
| IL-6 | WT vs C71G^+/-^ | | 0.9975 | | |
| IL-10 | WT vs C71G^+/-^ | | 0.98 | | |
| CCL5 | WT vs C71G^+/-^ | | 0.9682 | | |
| TNF-α | WT vs C71G^+/-^ | | 0.9982 | | |
| **Cytokine** | **Other comparison for 24h LPS iMGs** | | ***P value*** | | |
| IL-6 | WT vs C71G^+/-^ | | 0.9137 | | |
| IL-10 | WT vs C71G^+/-^ | | 0.7668 | | |
| CCL5 | WT vs C71G^+/-^ | | >0.9999 | | |
| TNF-α | WT vs C71G^+/-^ | | >0.9999 | | |
| **Supp. Figure 3** | | | | | |
| **Cytokine** | **Other comparison for WT iMGs** | | **P value** | | |
| IL-6 | Untreated vs 6h LPS | | 0.3593 | | |
| IL-6 | 6h LPS vs 24h LPS | | 0.0125 | | |
| IL-10 | 6h LPS vs 24h LPS | | 0.0024 | | |
| CCL5 | 6h LPS vs 24h LPS | | 0.0111 | | |
| TNF-α | Untreated vs 24h LPS | | 0.2564 | | |
| TNF-α | 6h LPS vs 24h LPS | | <0.0001 | | |
| **Cytokine** | **Comparison for M114T^+/-^ iMGs** | | **P value** | | |
| IL-6 | Untreated vs 6h LPS | | 0.2422 | | |
| IL-6 | Untreated vs 24h LPS | | <0.0001 | | |
| IL-6 | 6h LPS vs 24h LPS | | <0.0001 | | |
| IL-10 | Untreated vs 6h LPS | | <0.0001 | | |
| IL-10 | Untreated vs 24h LPS | | <0.0001 | | |
| IL-10 | 6h LPS vs 24h LPS | | 0.9976 | | |
| CCL5 | Untreated vs 6h LPS | | 0.0573 | | |
| CCL5 | Untreated vs 24h LPS | | <0.0001 | | |
| CCL5 | 6h LPS vs 24h LPS | | 0.0002 | | |
| TNF-α | Untreated vs 6h LPS | | <0.0001 | | |
| TNF-α | Untreated vs 24h LPS | | 0.4064 | | |
| TNF-α | 6h LPS vs 24h LPS | | <0.0001 | | |
| **Cytokine** | **Comparison for M114T^+/+^ iMGs** | | **P value** | | |
| IL-6 | Untreated vs 6h LPS | | 0.1224 | | |
| IL-6 | Untreated vs 24h LPS | | <0.0001 | | |
| IL-6 | 6h LPS vs 24h LPS | | <0.0001 | | |
| IL-10 | Untreated vs 6h LPS | | <0.0001 | | |
| IL-10 | Untreated vs 24h LPS | | <0.0001 | | |
| IL-10 | 6h LPS vs 24h LPS | | 0.9987 | | |
| CCL5 | Untreated vs 6h LPS | | 0.0728 | | |
| CCL5 | Untreated vs 24h LPS | | <0.0001 | | |
| CCL5 | 6h LPS vs 24h LPS | | <0.0001 | | |
| TNF-α | Untreated vs 6h LPS | | <0.0001 | | |
| TNF-α | Untreated vs 24h LPS | | 0.0407 | | |
| TNF-α | 6h LPS vs 24h LPS | | <0.0001 | | |
| **Cytokine** | **Other comparison for Untreated iMGs** | | **P value** | | |
| IL-6 | WT vs M114T^+/-^ | | 0.9842 | | |
| IL-6 | WT vs M114T^+/+^ | | 0.266 | | |
| IL-6 | M114T^+/-^ vs M114T^+/+^ | | 0.8712 | | |
| IL-10 | WT vs M114T^+/-^ | | 0.6207 | | |
| IL-10 | WT vs M114T^+/+^ | | 0.0234 | | |
| IL-10 | M114T^+/-^ vs M114T^+/+^ | | 0.3153 | | |
| CCL5 | WT vs M114T^+/-^ | | 0.7881 | | |
| CCL5 | WT vs M114T^+/+^ | | 0.8712 | | |
| CCL5 | M114T^+/-^ vs M114T^+/+^ | | 0.5879 | | |
| TNF-α | WT vs M114T^+/-^ | | 0.979 | | |
| TNF-α | WT vs M114T^+/+^ | | 0.9783 | | |
| TNF-α | M114T^+/-^ vs M114T^+/+^ | | 0.9987 | | |
| **Cytokine** | **Other comparison for 6h LPS iMGs** | | **P value** | | |
| IL-6 | WT vs M114T^+/-^ | | 0.0444 | | |
| IL-6 | WT vs M114T^+/+^ | | 0.319 | | |
| IL-6 | M114T^+/-^ vs M114T^+/+^ | | 0.4762 | | |
| IL-10 | WT vs M114T^+/-^ | | 0.5994 | | |
| IL-10 | WT vs M114T^+/+^ | | 0.5604 | | |
| IL-10 | M114T^+/-^ vs M114T^+/+^ | | 0.9991 | | |
| CCL5 | WT vs M114T^+/-^ | | 0.7376 | | |
| CCL5 | WT vs M114T^+/+^ | | 0.8278 | | |
| CCL5 | M114T^+/-^ vs M114T^+/+^ | | 0.9807 | | |
| TNF-α | WT vs M114T^+/-^ | | 0.8716 | | |
| TNF-α | WT vs M114T^+/+^ | | 0.5694 | | |
| TNF-α | M114T^+/-^ vs M114T^+/+^ | | 0.6793 | | |
| **Cytokine** | **Other comparison for 24h LPS iMGs** | | **P value** | | |
| IL-6 | WT vs M114T^+/-^ | | 0.5524 | | |
| IL-6 | WT vs M114T^+/+^ | | 0.051 | | |
| IL-6 | M114T^+/-^ vs M114T^+/+^ | | 0.1991 | | |
| IL-10 | WT vs M114T^+/-^ | | 0.2077 | | |
| IL-10 | WT vs M114T^+/+^ | | 0.1823 | | |
| IL-10 | M114T^+/-^ vs M114T^+/+^ | | 0.9986 | | |
| CCL5 | WT vs M114T^+/-^ | | 0.925 | | |
| CCL5 | WT vs M114T^+/+^ | | 0.1861 | | |
| CCL5 | M114T^+/-^ vs M114T^+/+^ | | 0.3569 | | |
| TNF-α | WT vs M114T^+/-^ | | 0.9866 | | |
| TNF-α | WT vs M114T^+/+^ | | 0.7237 | | |
| TNF-α | M114T^+/-^ vs M114T^+/+^ | | 0.6969 | | |
| **Figure 4b** | | | | | |
| **Time (h)** | | | ***P value*** | | |
| 0 | | | >0.9999 | | |
| 1 | | | 0.9076 | | |
| 2 | | | 0.0327 | | |
| 3 | | | 0.0003 | | |
| 4 | | | <0.0001 | | |
| 5 | | | <0.0001 | | |
| 6 | | | <0.0001 | | |
| 7 | | | <0.0001 | | |
| 8 | | | <0.0001 | | |
| 9 | | | <0.0001 | | |
| 10 | | | <0.0001 | | |
| 11 | | | <0.0001 | | |
| 12 | | | <0.0001 | | |
| **Figure 4i** | | | | | |
| **Condition** | | **Comparison** | ***P value*** | ***t*** | ***df*** |
| AF-synap - / BafA - | | WT vs C71G^+/-^ | >0.9999 | 0.0517 | 12 |
| AF-synap + / BafA - | | WT vs C71G^+/-^ | 0.9566 | 0.4675 | 12 |
| AF-synap + / BafA + | | WT vs C71G^+/-^ | >0.9999 | 0.0394 | 12 |
| **Comparison** | | | ***P value*** | ***t*** | ***df*** |
| AF-synap - / BafA - vs AF-synap + / BafA - | | | <0.0001 | 7.759 | 12 |
| AF-synap - / BafA – vs AF-synap + / BafA + | | | 0.0099 | 3.653 | 12 |
| **Supp. Figure 7** | | | | | |
| **Condition** | | **Comparison** | ***P value*** | ***t*** | ***df*** |
| AF-synap - / BafA - | | WT vs M114T^+/-^ | 0.9997 | 0.0859 | 18 |
| AF-synap - / BafA - | | WT vs M114T^+/+^ | >0.9999 | 0.0390 | 18 |
| AF-synap + / BafA - | | WT vs M114T^+/-^ | 0.9088 | 0.6094 | 18 |
| AF-synap + / BafA - | | WT vs M114T^+/+^ | 0.9057 | 0.6172 | 18 |
| AF-synap +/ BafA + | | WT vs M114T^+/-^ | 0.9998 | 0.0741 | 18 |
| AF-synap +/ BafA + | | WT vs M114T^+/+^ | >0.9999 | 0.0129 | 18 |
| **Comparison** | | | ***P value*** | ***t*** | ***df*** |
| AF-synap - / BafA - vs AF-synap + / BafA - | | | <0.0001 | 10.58 | 18 |
| AF-synap - / BafA - vs AF-synap + / BafA + | | | 0.0005 | 4.736 | 18 |
| **Figure 7e** | | | | | |
| **Genotype** | | **Comparison** | ***P value*** | ***q*** | ***df*** |
| WT | | Untreated vs Rapamycin | 0.7555 | 0.6527 | 30 |
| C71G^+/-^ | | Untreated vs Rapamycin | 0.1178 | 1.928 | 30 |
| C71G^+/-^ | | Untreated vs BafA | 0.0135 | 2.898 | 30 |
