# Supplementary material for: Expression of ALS-PFN1 impairs vesicular degradation in iPSC-derived microglia
Source: Nat Commun. 2024 Mar 20;15:2497. doi: 10.1038/s41467-024-46695-w (PMC10954694; doi:10.1038/s41467-024-46695-w)
Supplement: Supplementary file 11 — Reporting Summary [file 41467_2024_46695_MOESM11_ESM.pdf]

nature portfolio

Corresponding author(s): Daryl Bosco

Last updated by author(s): Jan 8, 2024

Reporting Summary

Nature Portfolio wishes to improve the reproducibility of the work that we publish. This form provides structure for consistency and transparency in reporting. For further information on Nature Portfolio policies, see our Editorial Policies and the Editorial Policy Checklist.

Statistics

For all statistical analyses, confirm that the following items are present in the figure legend, table legend, main text, or Methods section.

n/a	Confirmed	
☐	☒	The exact sample size (n) for each experimental group/condition, given as a discrete number and unit of measurement
☐	☒	A statement on whether measurements were taken from distinct samples or whether the same sample was measured repeatedly
☐	☒	The statistical test(s) used AND whether they are one- or two-sided<br>*Only common tests should be described solely by name; describe more complex techniques in the Methods section.*
☒	☐	A description of all covariates tested
☐	☒	A description of any assumptions or corrections, such as tests of normality and adjustment for multiple comparisons
☐	☒	A full description of the statistical parameters including central tendency (e.g. means) or other basic estimates (e.g. regression coefficient) AND variation (e.g. standard deviation) or associated estimates of uncertainty (e.g. confidence intervals)
☐	☒	For null hypothesis testing, the test statistic (e.g. F, t, r) with confidence intervals, effect sizes, degrees of freedom and P value noted<br>*Give P values as exact values whenever suitable.*
☒	☐	For Bayesian analysis, information on the choice of priors and Markov chain Monte Carlo settings
☒	☐	For hierarchical and complex designs, identification of the appropriate level for tests and full reporting of outcomes
☐	☒	Estimates of effect sizes (e.g. Cohen's d, Pearson's r), indicating how they were calculated

Our web collection on statistics for biologists contains articles on many of the points above.

Software and code

Policy information about availability of computer code

Data collection	Data was not collected with costume algorithm or code. Images were collected with AF6000 Leica Software v3.1.0 (Leica Microsystems) or Gen5 software v3.03 (Biotek). Flow cytometry data was collected with a MACSQuant VYB Flow Cytometer. Capillary-based western blot data was collected using a WES 3.8.21 instrument (ProteinSimple, BioTechne).

Data analysis	Live cell images were analyzed with Gen5 v3.03 software (Biotek). Fixed microscope images were analyzed using ImageJ/Fiji v2.3.0 and CellProfiler v4.2.5. For analysis in Figure 4j, a previously published costume code was used (https://visikol.com/blog/2018/11/29/blog-post-loading-and-measurement-of-volumes-in-3d-confocal-image-stacks-with-imagej/). The custom code used to draw concentric circles for analysis in Figure 5 e-g is located in the supplemental information.<br><br>RNAseq and PCA analysis were performed in R studio (RStudio 2022.02.0+443.pro2 with R version 4.0.4). Quantitative proteomics analysis was performed in Proteome Discoverer 2.1.1.21 (Thermo Fisher Scientific Inc.) and Scaffold v4.4.4 (Proteome Software, Inc.). Functional enrichment analysis used Metascape v3.5 (https://metascape.org) and Enrichr (https://maayanlab.cloud/Enrichr/, build 06.2023). For densitometry analysis Image Studio v 3.1.4 (Li-Cor) was utilized. Capillary-based western blot data was analyzed using Compass software v6.3.0 (Biotechne).<br><br>Flow cytometry data was analyzed using FlowJo v10.8.1 (BD Life Sciences).<br><br>NMR studies used NMRPipe v11.5 rev 2023.105.21.31, Sparky (NMRFAM-Sparky build_04-22-2021), and Matlab R2019a 64-bit (9.6.0.1072779) software.<br><br>Statistical analyses were calculated using GraphPad Prism v9.3.1.

For manuscripts utilizing custom algorithms or software that are central to the research but not yet described in published literature, software must be made available to editors and reviewers. We strongly encourage code deposition in a community repository (e.g. GitHub). See the Nature Portfolio guidelines for submitting code & software for further information.

Data

Policy information about availability of data

All manuscripts must include a data availability statement. This statement should provide the following information, where applicable:

- Accession codes, unique identifiers, or web links for publicly available datasets
- A description of any restrictions on data availability
- For clinical datasets or third party data, please ensure that the statement adheres to our policy

RNAseq data was deposited at GEO under accession number GSE254069. Mass spectrometry data has been deposited to the ProteomeXchange Consortium via the PRIDE partner repository with the dataset identifier PXD038943. Other data has been made available in a Source Sata file.

Research involving human participants, their data, or biological material

Policy information about studies with human participants or human data. See also policy information about sex, gender (identity/presentation), and sexual orientation and race, ethnicity and racism.

Reporting on sex and gender	The human induced pluripotent stem cell lines used (KOLF2.1J, WTC11) in this study are from male donors.
Reporting on race, ethnicity, or other socially relevant groupings	This study did not involve human participants. The iPSC lines used are from a white man of European ancestry (KOLF2.1J) and a man of Japanese ancestry (WTC11).
Population characteristics	This study did not involve human participants.
Recruitment	Human participants were not recruited in this study.
Ethics oversight	Human iPSC cell lines used in this study were de-identified and are not subject to Institutional Review Board oversight.

Note that full information on the approval of the study protocol must also be provided in the manuscript.

Field-specific reporting

Please select the one below that is the best fit for your research. If you are not sure, read the appropriate sections before making your selection.

☒ Life sciences ☐ Behavioural & social sciences ☐ Ecological, evolutionary & environmental sciences

For a reference copy of the document with all sections, see nature.com/documents/nr-reporting-summary-flat.pdf

Life sciences study design

All studies must disclose on these points even when the disclosure is negative.

Sample size	No statistical method was used to predetermine sample size. Sample sizes for all experiments were determined based on previous publications as follows.<br><br>For all experiments performed using iPSCs and iMGs, we based the number of biological replicates (independent differentiations) on previous literature using iPSC-derived microglia including Brownjohn et al., Stem Cell Reports 2018. For analyses in which statistics were reported, a minimum of 3 independent differentiations were generated.

For p62/LC3 puncta analyses, we based a minimum number of analyzed cells on a paper published by Runwal et al. in Scientific Reports 2019 in which they showed IF staining for LC3 in HeLa cells. They quantified LC3 puncta average size and number per cell from 3 experiments with >25 cells per condition. In this study, >25 cells per condition were analyzed from 4 independent differentiations for a total of 144 WT cells and 143 PFN1 C71G+/- cells.

For analyses using the HMC3 cell lines, we based a minimum number of 3 independent experiments on previous literature studying HMC3 (ex: Baek et al., Scientific Reports 2021). We increased the number of independent experiments to 5 to better account for statistical variation.

For animal studies, we analyzed at least four animals per genotype with at least 2 male and 2 female animals. This is insufficient to draw statistical conclusions on sex-related differences, but can be sufficient to draw overall conclusions based on previous literature (ex: Yang et al., PLoS One 2022).

Data exclusions: For the p62 and LC3 colocalization analysis, cells that had no detected p62 puncta were excluded (3 out of 147 WT cells, 1 out of 144 PFN1 C71G+/- cells)

Replication: All replication attempts were successful. Experiments were repeated multiple times as indicated in the Methods and/or Figure legends. Only reproducible data is included in the manuscript.

Randomization: Randomization is not relevant in this study. Cell lines and animals were grouped according to genotype.

Blinding: Groups were allocated based on genotype of the cell line or animal, so blinding was not relevant to data collection.

For immunofluorescence analyses, investigators were blinded to genotype during data collection and analysis.

For western blot, NMR, RNAseq, qPCR, proteomics, flow cytometry, ELISA, and phagocytosis assay experiments, investigators were not blinded to genotype during data collection or analysis. For the phagocytosis experiments, blinding was not relevant as the fields of view were pre-selected automatically in the Gen5 software based on location within the well without user input that could be subject to bias. For the other experiments (i.e., Western blot analyses, omics), the input was representative of the entire sample.

Reporting for specific materials, systems and methods

We require information from authors about some types of materials, experimental systems and methods used in many studies. Here, indicate whether each material, system or method listed is relevant to your study. If you are not sure if a list item applies to your research, read the appropriate section before selecting a response.

Materials & experimental systems

n/a	Involved in the study	
☐	☒	Antibodies
☐	☒	Eukaryotic cell lines
☒	☐	Palaeontology and archaeology
☐	☒	Animals and other organisms
☒	☐	Clinical data
☒	☐	Dual use research of concern
☒	☐	Plants

Methods

n/a	Involved in the study	
☒	☐	ChIP-seq
☐	☒	Flow cytometry
☒	☐	MRI-based neuroimaging

Antibodies

Antibodies used:

Protein Species Clone Supplier Catalog # Lot # Application Dilution
P2RY12 Rabbit polyclonal Sigma-Aldrich HPA014518 000036809 IF 1:50
TMEM119 Rabbit polyclonal Sigma-Aldrich HPA051870 000004428 IF 1:100
IBA1 Rabbit polyclonal Wako Chemical USA NC9288364 WTF4691
IF 1:350 (cells), 1:500 (mouse tissue)
PFN1 Mouse 1D5 GeneTex GTX83903 822301969 IF 1:100-350
OCT4 Mouse #653108 R&D Systems MAB17591-SP CEBN0221081 IF 1:1000
SOX2 Mouse #245610 R&D Systems MAB2018-SP KGQ0422042 IF 1:1000
CD68 Rabbit EPR20545 Abcam ab213363 GR3266939-13 IF 1:100
TBC1D15 Rabbit polyclonal Sigma-Aldrich HPA013388 000034860 IF, WB 1:100 (IF), 1:500 (WB)
LAMP1 Mouse H4A3 Developmental Studies Hybridoma Bank H4A3 Produced 01/28/21 IF 1:100
EEA1 Mouse 14/EEA1 BD Bioscience 610456 5357682 IF 1:100
RAB7 Rabbit D95F2 Cell Signaling Technology 9367T 3 IF, WB 1:100 (IF), 1:1000 (WB)
TOMM20-AF647 Mouse EPR15581-39 Abcam ab309166 1053061-2 IF 1:150
p62 Guinea Pig polyclonal PROGEN Biotechnik GP62-C FAK15149-02 IF, WB 1:100 (IF), 1:1000 (WB)
LC3 Mouse 4E12 MBL International M152-3 058 IF 1:200
PLIN2 Guinea Pig polyclonal Fitzgerald Industries 20R-AP002 0000062884 IF 1:200
PFN1 Rabbit polyclonal Sigma-Aldrich P7749 IF, WB 1:100 (IF), 1:1000 (WB)
LC3 Rabbit polyclonal Sigma-Aldrich L7543 0000129656 WB 1:1000
CTSD Rabbit polyclonal Cell Signaling Technology 2284 2 WB 1:1000
VDAC1 Mouse 20B12AF2 Abcam ab14734 2101034688 WB 1:1000

RASD2 Rabbit polyclonal Abclonal 17367 WB 1:1000
GAPDH Mouse GAPDH-71.1 Sigma-Aldrich G8795 068M4857V WB 1:10000
GAPDH Rabbit polyclonal Sigma-Aldrich G9545 0000148622 WB 1:10000
GFAP Mouse G-A-5 Sigma-Aldrich G3893 0000181213 IF 1:500
CD68 Rat FA-11 BioRad MCA1957 155083 IF 1:200
PFN1 Rabbit polyclonal Cell Signaling Technology 3237S SimpleWestern WB 1:25
GAPDH Rabbit polyclonal NovusBio NB300-322 SimpleWestern WB 1:5000
Alexa Fluor® 488 AffiniPureTM Donkey Anti-Rabbit IgG (H+L) Donkey polyclonal Jackson ImmunoResearch 711-545-152 165045 IF 1:2000
Alexa Fluor® 488 AffiniPureTM Donkey Anti-Mouse IgG (H+L) Donkey polyclonal Jackson ImmunoResearch 715-545-150 165936 IF 1:2000
Alexa Fluor® 488 AffiniPureTM Goat Anti-Guinea Pig IgG (H+L) Goat polyclonal Jackson ImmunoResearch 106-545-003 156732 IF 1:2000
Cy3 AffiniPure™ Donkey Anti-Rabbit IgG (H+L) Donkey polyclonal Jackson ImmunoResearch 711-165-152 163463 IF 1:2000
Cy3 AffiniPure™ Donkey Anti-Mouse IgG (H+L) Donkey polyclonal Jackson ImmunoResearch 715-165-151 IF 1:2000
Cy3 AffiniPure™ Fab Fragment Donkey Anti-Rat IgG (H+L) Donkey polyclonal Jackson ImmunoResearch 712-167-003 158835 IF 1:2000
Alexa Fluor® 647 AffiniPure™ Donkey Anti-Rabbit IgG (H+L) Donkey polyclonal Jackson ImmunoResearch 711-605-152 160477 IF 1:2000
Alexa Fluor® 647 AffiniPure™ Donkey Anti-Mouse IgG (H+L) Donkey polyclonal Jackson ImmunoResearch 715-605-151 162532 IF 1:2000
IRDye® 800CW Goat anti-Rabbit IgG Secondary Antibody Goat polyclonal LI-COR 926-32211 D11215-03 WB 1:10000
IRDye® 680RD Donkey anti-Mouse IgG Secondary Antibody Donkey polyclonal LI-COR 926-68072 D11116-05 WB 1:10000
IRDye® 680LT Donkey anti-Guinea Pig IgG Secondary Antibody Donkey polyclonal LI-COR 926-68030 D00916-09 WB 1:10000

Validation

anti-P2RY12 was verified by Sigma-Aldrich using orthogonal RNA-seq and has been used in multiple papers to label hiPSC-derived microglia cells (ex: Bassil et al., Nature Communications 2021)
anti-TMEM119 was verified by Sigma-Aldrich by co-staining with an independent anti-TMEM119 antibody (HPA052650) and by orthogonal RNA-seq. This antibody has been used in multiple publications to label micrsoglia i n human brain tissue (ex: Muñoz-Castro et al., Methods Mol Biol 2023).
anti-Iba1 lists immunocytochemistry as a validated application on their product site. This antibody has been used in multiple publications to label microglia in human brain tissue (ex: Zhao et al., Cell 20A20)
anti-PFN1 (Genetex) was verified by detection of overexpression of PFN1 in HEK293T cells. The antibody also detected a decrease in expression of PFN1 in HMC3 cells in which PFN1 was knocked down (Supp Fig 5) in this study (the knockdown of PFN1 was also confirmed via Western blot with a different PFN1 antibody in this study).
anti-OCT4 lists immunocytochemistry as a validated application and provides an example of staining in BG01V human stem cells. One example of this antibody being used to label human embryonic stem cells is in Vlahova et al., Sci Rep 2019.
anti-SOX2 lists immunocytochemistry as a validated application and provides an example of staining in BG01V human stem cells. This antibody has been used in multiple publications to label human embryonic stem cells (ex:
Souralova et al., Int J Mol Sci 2022)
anti-CD68 has ICC/IF listed as a tested application on the Abcam listing. In one published example (Pediaditakis et al., iScience 2022), this antibody is used to track activation of a human microglial cell line (HMC3) in their system.
anti-TBC1D15 has immunofluorescence listed as a validated application on their product page. As a Prestige Antibody, the vendor states the antibody has been tested and validated through an IHC tissue array of 44 normal human tissues and 20 common cancer type tissues and a protein array of 364 human recombinant protein fragmeants. In one publication (Chai et al., Brain Res 2020), the antibody is KO validated in the human myeloid U937 cell line.
anti-LAMP1 has immunofluorescence listed as a recommended application on the product page. This antibody has been used in multiple publications to label lysosomes in hiPSC-derived cells (ex: Uusi-Rauva et al., Int J Mol Sci 2017).
anti-EEA1 has a statement that immunofluorescence is a tested application on their product page and an example image of staining on human smooth muscle tissue. This antibody has been used in publications to label endosomes in hiPSC-derived cells (ex: Fong et al., J Biol Chem 2018).
anti-Rab7 lists both WB and IF as product applications on the product page with an example WB image on multiple human cell lines and an example IF image on a human melanocyte line. One example of a published paper using Rab7 for both WB and IF in a human cell line (SH-SY5Y) is Iketani et al., Med Gas Res 2023.
anti-TOMM20-647 lists ICC/IF as a tested application on the product page and provides an example IF image on HeLa cells. Our lab compared staining patterns with a different TOMM20 antibody (ProteinTech 66777-1) and found they appeared the same.
anti-p62 lists both IHC and WB as tested applications on the product page. This antibody is used in many publications for WB and IF analysis in human cells (ex: Schmitt et al., EMBO Rep 2022).
anti-LC3 (MBL) lists ICC as a tested application on the product site. One example of a publication using this antibody for IF on human ARPE-19 cells is Wilhelm et al., EMBO J 2022.
anti-PLIN2 lists IHC-F as a tested application on the product page. One example of a publication using this antibody for IF on a human monocytic THP-1 line is Mulye et al., PLoS One 2018.
anti-PFN1 (Sigma) lists both IF and WB as applicable techniques on the product page. One published example of using this antibody to recognize PFN1 in human cell lines (LCLs) via WB is Freischmidt et al., BMC Neurosci 2015. Our lab compared the staining pattern of this antibody and another PFN1 antibody (Genetex) for IF on our iMGs and found that they appeared the same.
anti-LC3 (Sigma) lists WB as a validated application and also state that enhanced validation was performed via functional assay. One example of a publication using this antibody to track autophagy in human stem cells is Cen et al., Stem Cell Res Ther 2019.
anti-CTSD lists WB as a tested application on the product page. One example of a publication using it for WB on human monocytes is Kabelitz et al., Sci Rep 2022.
anti-VDAC1 lists WB as a tested application and states that it has been KO validated on the product page. One example of a publication using this antibody to track mitochondria levels in a human cell line (SH-SY5Y) is Risiglione et al., Life (Basel) 2022.
anti-RASD2 lists WB as a tested application on its product page. Our lab used this antibody to confirm changes found through RNA-seq on our samples, thereby validating it.
anti-GAPDH (Sigma G8795) lists WB as a tested application on the product page. This antibody has been used in many publications for WB on human iPSC model systems (ex: Mubariz et al., Front Neurosci 2023)
anti-GAPDH (Sigma G9545) lists WB as a tested application on the product page. One example of a publication using it for WB on human monocyte-derived macrophages is Hammoud et al., Mol Oncol 2022.

anti-GFAP lists immunofluorescence as a suitable application on the product page. This antibody is used in many publications as a marker for astrocytes in mouse tissue (ex: Beyer et al., Front Neurosci 2021).
anti-CD68 (BioRad) lists IF on tissue as a verified application on the product page. This antibody is used in many publications as a marker for phagocytosis in mouse tissue microglia (ex: Unger et al., Alzheimer's & Dementia 2018).
anti-PFN1 (Cell Signaling Technology) lists WB as a suggested application. One recent publication using this antibody for WB on mouse tissue is Baharani et al., Cell Mol Neurobiol 2022.
anti-GAPDH (NovusBio) lists WB and Simple Western as validated applications on the product page, as well as a statement that the antibody has been knockdown validated. One publication that uses this antibody for Simple Western WB on mouse tissue is Presa et al. J Clin Invest. 2021.

Eukaryotic cell lines

Policy information about cell lines and Sex and Gender in Research

Cell line source(s)

All KOLF2.1J iPSC lines (WT and PFN1 mutants) were obtained from The Jackson Laboratory for Genomic Medicine (Farmington, CT) and were generated as described in the Methods section and in the publication cited in that section. All KOLF2.1J iPSC lines used are described in Supp. Fig 1. The KOLF2.1J cell line originated from a male subject of European descent.

The WTC cell line with the hNIL transcription factor cassette was provided by the lab of Dr. Michael Ward (NIH). The WTC11 cell line originated from a male subject of Japanese descent.

The HMC3 line was purchased from ATCC (commercial).

Authentication

All iPSC lines were karyotyped, genotyped using Sanger sequencing, and tested for pluripotency markers using immunofluorescence staining (Supp. Fig 1).

The HMC3 line was purchased from ATCC. On their website, they state that their product is strongly positive for the microglia/macrophage marker IBA1, positive for endotoxin receptor CD14, but negative for astrocyte marker GFAP. They further state that markers of activated microglia (MHCII, CD68. CD11b) are negative at baseline, but upregulated upon activation by IFN-gamma (10 ng/mL, 24h). Of the above, our lab confirmed expression of IBA1 through immunofluorescence staining.

Mycoplasma contamination

All cell lines used in this study tested negative for mycoplasma contamination.

Commonly misidentified lines
(See ICLAC register)

No misidentified lines were used.

Animals and other research organisms

Policy information about studies involving animals; ARRIVE guidelines recommended for reporting animal research, and Sex and Gender in Research

Laboratory animals

Wild-type C57BL/6J mouse embryos (E17.5) or pups (P18-21) were used for isolation of primary cortical neurons or purification of synaptosomes respectively.

3-month-old C57BL/6J-Pfn1em4Lutzy/J (#030313) mice were used for analysis in Figure 5.
8-week-old animals of the same strain were used for Simple Western assays (Supp Fig 12).
~600-day-old animals (between P593 and P628) of the same strain were tested for motor function via rotarod and grip strength measurements (Supp Fig 12). Those same animals were euthanized and their tissue processed for immunostaining (Supp Fig 13).

All experimental procedures involving mice were performed in accordance with the guidelines of Institutional Animal Care and Use Committee (IACUC) of the University of Massachusetts Chan Medical School. Mice were housed in rooms that are controlled for a 12hr/12hr light/dark cycle with an ambient temperature of 68-72 degrees F and relative humidity between 30%-70%.

Wild animals

This study did not involve wild animals

Reporting on sex

For each experiment using mice, numbers of male and female of each genotype (WT, PFN1 C71G+/-) were reported in the methods and/or the figure legend. Different symbols were used for data points based on sex of the animal (open symbols for male, closed for female). Desegregated male and female data are reported in the Source Data file.

Findings are not sex-specific. Sex was considered in study design when possible by including as equal numbers of male and female animals per experiment as possible.

Field-collected samples

This study did not involve field-collected samples.

Ethics oversight

The Institutional Animal Care and Use Committee (IACUC) at the University of Massachusetts Chan Medical School approved all experiments using animals.

Note that full information on the approval of the study protocol must also be provided in the manuscript.

Plants

Seed stocks | *Report on the source of all seed stocks or other plant material used. If applicable, state the seed stock centre and catalogue number. If plant specimens were collected from the field, describe the collection location, date and sampling procedures.*

Novel plant genotypes | *Describe the methods by which all novel plant genotypes were produced. This includes those generated by transgenic approaches, gene editing, chemical/radiation-based mutagenesis and hybridization. For transgenic lines, describe the transformation method, the number of independent lines analyzed and the generation upon which experiments were performed. For gene-edited lines, describe the editor used, the endogenous sequence targeted for editing, the targeting guide RNA sequence (if applicable) and how the editor was applied.*

Authentication | *Describe any authentication procedures for each seed stock used or novel genotype generated. Describe any experiments used to assess the effect of a mutation and, where applicable, how potential secondary effects (e.g. second site T-DNA insertions, mosiacism, off-target gene editing) were examined.*

Flow Cytometry

Plots

Confirm that:

☒ The axis labels state the marker and fluorochrome used (e.g. CD4-FITC).

☒ The axis scales are clearly visible. Include numbers along axes only for bottom left plot of group (a 'group' is an analysis of identical markers).

☐ All plots are contour plots with outliers or pseudocolor plots.

☐ A numerical value for number of cells or percentage (with statistics) is provided.

Methodology

Sample preparation | iMG cells were pre-treated with or without 100 nM bafilomycin A for 30 min at 37 degrees C. They were then incubated at 10 degrees C for 10 min and subsequently fed with AF546-labeled synaptosomes. Cells were then centrifuged at 270 x g for 3 min at 10 degrees C followed by an incubation at 37 degrees C for 15 min. Cells were dissociated with Accutase for 5 min at 37 degrees C and collected into DPBS in microcentrifuge tubes that had been pre-coated with 5% BSA/PBS to prevent sticking of the cells to the plastic. Cells were centrifuged at 300 x g for 5 min before being resuspending in 100 uL of 1% FBS/DPBS for data collection on a flow cytometer.

Instrument | MACSQuant VYB Flow Cytometer

Software | FlowJo v10.8.1 (BD Life Sciences)

Cell population abundance | A subpopulation based on Forward scatter (FSC) and side scatter (SSC) was gated upon to select for intact cells. This subpopulation contained around 50-70% of all events collected across the different samples.

Gating strategy | A polygonal gate was drawn on SSC- area (A) and FSC-A to select for intact cells. SSC-A values ranged from around 15K-140K and FSC-A values ranged from around 15K-150K. An example of this gating strategy is included in the Source Data file (Supp Fig. 10A, B).

☒ Tick this box to confirm that a figure exemplifying the gating strategy is provided in the Supplementary Information.
